# Prescribing Biologic Immune-Modifying Therapies for Patients with a History of Cancer: A Cross-Specialty Review

**DOI:** 10.3390/curroncol33080445

**Published:** 2026-07-24

**Authors:** Stephanie Bowe, Seamus O’Reilly, Michelle Murphy, Anne O’Mahony, Sinead Harney, Akbar Zulquernain, John Bourke

**Affiliations:** 1 Dermatology Department, South Infirmary Victoria University Hospital, T12 X23H Cork, Ireland; drstephaniebowe@gmail.com (S.B.); drjfbourke@gmail.com (J.B.); 2 Department of Medical Oncology, Cork University Hospital, T12 DC4A Cork, Ireland; 3 Cancer Trials Ireland, 123 St. Stephens Green, D02 H903 Dublin, Ireland; 4 Respiratory Department, Cork University Hospital, T12 DC4A Cork, Ireland; anne.omahony5@hse.ie; 5 Rheumatology Department, Cork University Hospital, T12 DC4A Cork, Ireland; sinead.harney@hse.ie; 6 Gastroenterology Department, Cork University Hospital, T12 DC4A Cork, Ireland; syed.zulquernain@hse.ie

**Keywords:** malignancy, biologic, DMARD, psoriasis, rheumatoid arthritis, Crohn’s disease, asthma, registry data

## Abstract

The rapid expansion of biologic therapies for immune-mediated inflammatory diseases has raised significant clinical concerns regarding malignancy risk, particularly for patients with a history of cancer. This narrative review explores the safety of targeted therapies across dermatology, rheumatology, respiratory medicine, and gastroenterology to guide clinicians in these therapeutic dilemmas. We conducted a non-systematic review of the literature, prioritising longitudinal registry and real-world cohort data over clinical trials to better capture malignancy outcomes with long latency periods. Results indicate that tumour necrosis factor (TNF) inhibitors, which have the most extensive evidence base, do not consistently demonstrate an increased risk of overall incident malignancy or recurrence across specialties. Newer agents, including interleukin (IL)-17 and IL-23 inhibitors, show reassuring safety profiles in both trial and registry data. While dupilumab is associated with the potential diagnostic ‘unmasking’ of pre-existing cutaneous T-cell lymphoma, overall cancer rates remain stable among users. Most clinical guidelines support an individualised, multidisciplinary approach involving oncology consultation. We conclude that biologic agents can be used cautiously in certain patients with a history of malignancy following multidisciplinary discussion; however, in those with active malignancy there are no meaningful data that exist. Most evidence is heterogenous and excludes patients with a history of malignancy. Future management requires validated decision frameworks and mandatory participation in real-world patient co-created registries which leverage developing artificial intelligence tools to refine long-term safety assessments.

## Simple Summary

Biologic therapies have revolutionised the treatment of chronic inflammatory diseases such as psoriasis, arthritis, and asthma. However, their immunosuppressive effects have led to concerns about the risk of cancer promotion in patients with prior or active cancer illness. Reassurance for patients and prescribers is compounded by the exclusion of patients with cancer from pivotal clinical trials of these agents. This review collates large-scale health databases and real-world evidence to better understand these risks. These available data demonstrate that biologic agents can be used with caution in certain patients with a history of malignancy following multidisciplinary discussion; however, in those with active malignancy there is limited evidence. The majority of evidence is heterogenous and excludes patients with a history of malignancy. Such information is crucial for helping doctors and patients make informed decisions about treatment. Greater reassurance would be possible with more long-term follow-up and greater use of inclusive, patient co-created real-world registries which leverage developing artificial intelligence tools to monitor more diverse patient populations.

## 1. Introduction

**Figure 1:**
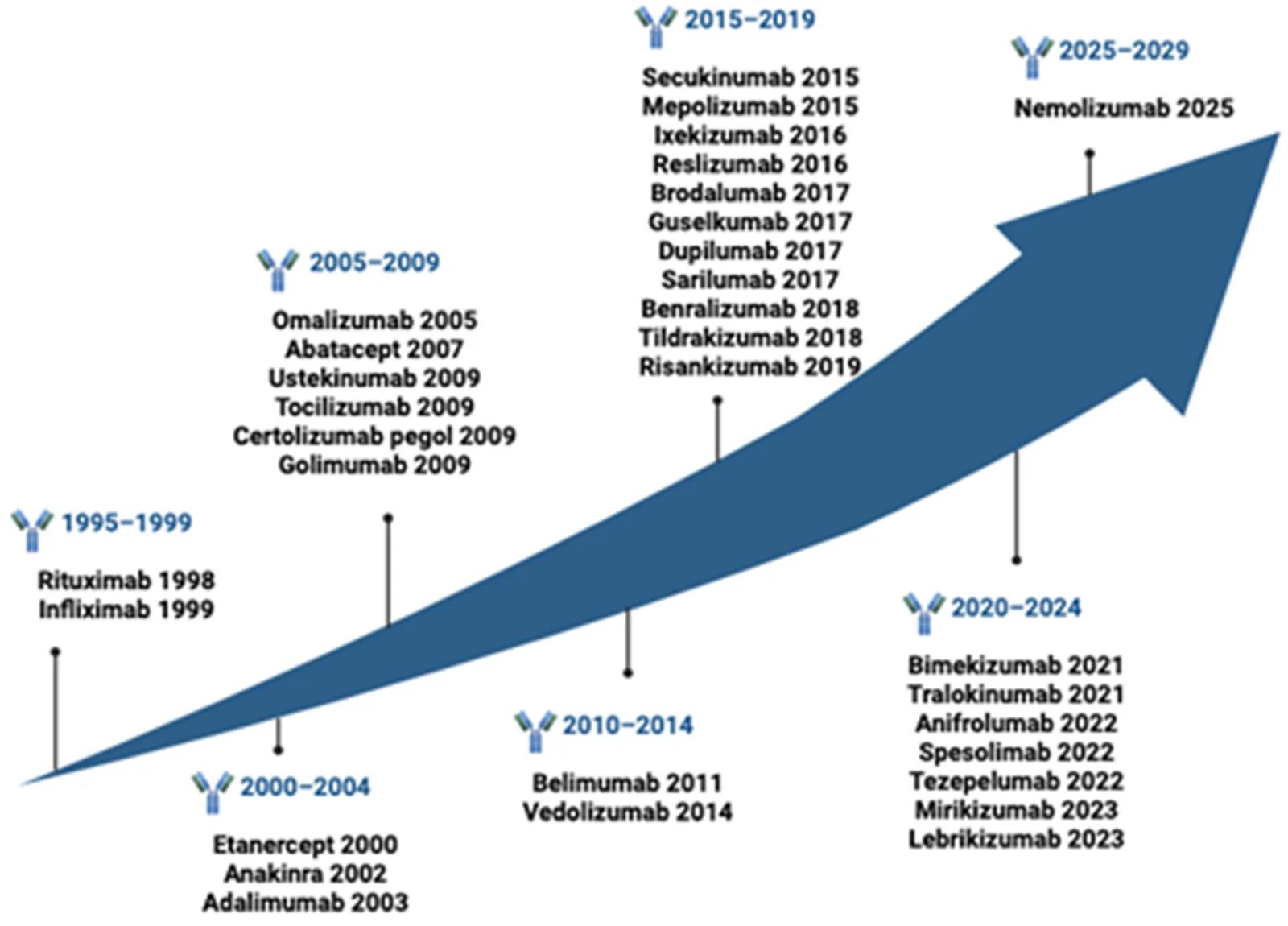
The increasing number of biologic agents approved in Ireland grouped in 5-year intervals. The blue arrow represents this substantial rise in biologic medication approvals.

Historically, oncology and immune-mediated inflammatory disease therapeutics have been aligned through the use of cytotoxic agents such as cyclophosphamide and methotrexate in both disease types. This interface became more nuanced in the past 5 decades with the Nobel prize-winning development of monoclonal antibodies by Köhler and Milstein [1]. Since then, a transformative exponential rise has occurred in the armamentarium of monoclonal antibodies in both disease types (Figure 1 and Table 1). The authors of this review are from Ireland, and though Figure 1 and Table 1 represent Ireland-specific data, this is representative of medical specialties globally and is relevant to all specialists in oncology. 

B-cell depletion therapies such as rituximab have pivotal roles in both spheres, whereas disease-modifying antirheumatic drugs (DMARDs) are used in only one. Their potentially deleterious effects on anti-tumour immunity have raised concerns about an increased risk of malignancy in patients receiving them [2,3,4,5]. This concern is even greater in patients with a history of cancer, where an increased cancer relapse rate could be a bystander effect of an otherwise quality of life transforming therapy.

Cancer and immune-mediated inflammatory diseases are among the most common and increasing non-communicable diseases globally [6]. Prescribing DMARDs in patients with a prior malignancy can present clinical dilemmas. While the available evidence does not support a deleterious effect in patients with cancer, this evidence has shortcomings. Most studies include long-term survivors rather than patients with newly diagnosed cancer [7]. Patients with cancer were often excluded from pivotal studies. Furthermore, the range of available personalised therapies is expanding, precluding even expert panels from formulating universal recommendations [8]. Available median follow-up in many studies is also limited (4.52 years in one recent meta-analysis [9]). This limits reassurance for patients such as those with breast cancer, where delayed relapse (defined as occurring 5 years or more post-diagnosis) is common. Finally, to address the informational needs of patients in these therapeutic dilemmas, we need their insights. However, the patient voice is missing from many of the available studies in this area. In 2025 the first ever study of the perspectives of patients with rheumatoid arthritis and concomitant cancer was published [10].

In light of the above shortcomings, we established a multidisciplinary group to review this area. This article is a narrative, non-systematic review of the literature across dermatology, rheumatology, respiratory medicine, and gastroenterology. The literature search was conducted between September 2023 and July 2026 inclusive. Searches were performed in PubMed/MEDLINE, Google Scholar and Cochrane Library using terms including, but not limited to: biologic therapy, DMARD, malignancy, cancer risk, cancer recurrence, TNF-inhibitor, IL-17 inhibitor, IL-23 inhibitor, dupilumab, omalizumab, vedolizumab, psoriasis, rheumatoid arthritis, inflammatory bowel disease, asthma, and combinations thereof. Only English-language publications were reviewed. The search was supplemented by manual review of reference lists of key articles and relevant clinical guidelines. Publications were included if they reported on malignancy risk or recurrence in patients receiving biologic or targeted therapies for immune-mediated inflammatory diseases. Priority was given to longitudinal registry studies, large real-world cohort studies, systematic reviews, meta-analyses, and clinical trial extension data. Case reports and small series were considered where higher-level evidence was absent, particularly for patients with a prior malignancy. Studies were excluded if they did not report malignancy as an outcome, or if the study population was not relevant to the clinical question. Where evidence was conflicting, we discuss the discordant findings in context, with reference to study design, follow-up duration, sample size, and the inclusion or exclusion of patients with prior malignancy as potential sources of heterogeneity. Formal quality assessment of individual studies was not performed, consistent with the narrative review methodology; however, the strength and consistency of evidence across studies is described throughout (Table 2).

Randomised clinical trials and their extensions can support short-term safety signals but are frequently underpowered for malignancy endpoints and commonly exclude patients with recent cancer (Table 3). As a result, clinical trial data are usually most useful when they explicitly include patients with a prior malignancy or report longer follow-up in relevant subgroups (Table 2) [11]. Where clinical trials were included, we prioritised open-label extensions or studies with longer reporting periods over shorter trials (Table 2 and Table 3).

**Table 2:** Summary of Registry and Real-World Observational Data.

Registry/Study	Disease Entity	Study Design	Exposed vs. Comparator, n	Median Follow-Up	Prior Malignancy Included	Key Malignancy Findings (Hazard Ratio, Relative Risk, Incidence Rate, 95% Confidence Interval)
PSOLAR (Papp et al., 2012) [12]	Psoriasis	Nested case-control analysis within a longitudinal registry	12,090 enrolled; 252 malignancy cases matched to 1008 controls	Median 4.17 years	Not stated	Tumour necrosis factor inhibitor use for 12 months or longer was associated with increased lung cancer risk (odds ratio 1.54, 95% confidence interval 1.10–2.15; *p* = 0.01); shorter exposure and individual agents, including ustekinumab, showed no significant association.
British Society for Rheumatology Biologics Register (BSRBR) (Dixon et al., 2010) [13]	Rheumatoid arthritis	National prospective observational cohort	177 tumour necrosis factor inhibitor–treated vs. 117 conventional disease-modifying drug–treated patients	3.1 person-years (tumour necrosis factor inhibitor cohort) vs. 1.9 person-years (conventional therapy cohort)	Yes—all 293 patients had a prior malignancy	Incidence of recurrent malignancy: 25.3 per 1000 person-years (tumour necrosis factor inhibitor) vs. 38.3 per 1000 person-years (conventional therapy); age- and sex-adjusted incidence rate ratio 0.58 (95% confidence interval 0.23–1.43).
RABBIT (Strangfeld et al., 2010) [14]	Rheumatoid arthritis	National prospective cohort (German biologics register)	5120 patients enrolled; 122 had a prior malignancy	Mean time to malignancy onset: 25.0 months (tumour necrosis factor inhibitor), 14.8 months (anakinra), 17.4 months (no biologic exposure); no median reported	Yes—122 of 5120 patients	No significant difference in incident or recurrent malignancy across exposure groups. Incidence rate without prior cancer: 6.0 per 1000 person-years. In a nested case-control analysis, prior tumour necrosis factor inhibitor exposure did not differ between cancer cases and cancer-free controls (44 vs. 44; *p* = 1.0).
BIOBADADERM (Olivares-Guerrero et al., 2025) [15]	Psoriasis	Prospective multicentre cohort (Spanish registry)	4212 patients; 7590 treatment cycles; no untreated comparator arm	Mean treatment exposure 4.1 years (standard deviation 3.7); no median reported	Not stated	Adalimumab incidence rate for all adverse events: 614 per 1000 patient-years (95% confidence interval 591–637). Ixekizumab, secukinumab, and guselkumab were associated with lower risk of benign and malignant neoplasms than adalimumab (adjusted incidence rate ratios <1, individual values reported in source).
Anti-Rheumatic Therapy in Sweden/Danish Rheumatologic Database (ARTIS-DANBIO) (Hellgren et al., 2017) [16]	Spondyloarthritis	Nationwide prospective cohort, pooled Swedish and Danish biologics registers	8703 tumour necrosis factor inhibitor initiators vs. 28,164 biologic-naïve patients and 131,687 matched general-population comparators	Median 5.6 years (interquartile range 1.0–10.2)	Not stated	Relative risk of cancer in biologic-naïve patients vs. general population: 1.1 (95% confidence interval 1.0–1.2). Relative risk in tumour necrosis factor inhibitor initiators vs. biologic-naïve patients: 0.8 (95% confidence interval 0.7–1.0); consistent across ankylosing spondylitis and psoriatic arthritis subgroups.
TREAT Registry (Lichtenstein et al., 2014) [17]	Crohn disease	Prospective cohort	3420 infliximab-exposed vs. 2509 treated with other agents only (6273 enrolled total)	Mean 5.2 years (all patients)/7.6 years (currently active patients); no median reported	Not stated	No independent association between infliximab or immunosuppressant therapy and malignancy. Independent predictors of malignancy were age (hazard ratio 1.59 per 10 years; *p* < 0.001), disease duration (hazard ratio 1.64 per 10 years; *p* = 0.012), and smoking (hazard ratio 1.38; *p* = 0.045); infliximab alone was not significant (hazard ratio 0.59; *p* = 0.16).
ESPRIT Registry (Menter et al., 2017) [18]	Psoriasis	Ongoing 10-year international prospective observational registry	6051 patients; 23,660.1 patient-years of adalimumab exposure; no comparator arm	7-year interim analysis; no median reported	Not stated	Incidence rate for malignancy: 1.0 event per 100 patient-years; serious adverse events 4.4 per 100 patient-years; serious infections 1.0 per 100 patient-years.
Psonet (Garcia-Doval et al., 2018) [19]	Psoriasis	Nested case-control analysis pooling four national registries (Israel, Italy, Spain, United Kingdom/Ireland)	27,376 patients; 58,978 person-years pooled across registries	Not reported as a median; person-years of follow-up given per registry (e.g., Spain 9425.85; United Kingdom/Ireland 30,961; Italy 14,871.47; Israel 3719.67)	Not stated	Pooled adjusted odds ratio for cancer per year of cumulative biologic exposure: 1.02 (95% confidence interval 0.92–1.13); no individual registry showed a significantly increased risk.
Swedish Cancer Registry cohort (Askling et al., 2009) [20]	Rheumatoid arthritis	National cohort, Swedish Inpatient/Outpatient and Early Rheumatoid Arthritis Registers	6604 first-time tumour necrosis factor inhibitor initiators vs. 61,160 biologic-naïve, 5989 new methotrexate, and 1838 combination-therapy comparators	Median 3.6 years (maximum 8 years), based on 25,693 person-years among 6366 initiators	Not stated	240 first cancers observed; relative risk vs. biologic-naïve cohort 1.00 (95% confidence interval 0.86–1.15); similar relative risks against the other comparator cohorts, with no difference between agents beyond the first year of follow-up.
PsABio (Gossec et al., 2023) [21]	Psoriatic arthritis	Prospective observational, head-to-head comparison	439 ustekinumab vs. 456 tumour necrosis factor inhibitor (895 in the effectiveness analysis set; 934 in the safety set)	Fixed 3-year study duration per participant; no median reported	Not stated	With a 1-year lag applied, malignancies during years 2–3 occurred in 3 of 494 ustekinumab-treated (0.6%) and 4 of 557 tumour necrosis factor inhibitor–treated patients (0.7%).
EXCELS Study (Long et al., 2014) [22]	Asthma	Prospective observational cohort	5007 omalizumab-treated vs. 2829 non-omalizumab comparators	Approximately 5 years in both cohorts	Not stated	Hazard ratio for all malignancies (omalizumab vs. comparator): 1.09 (95% confidence interval 0.87–1.38); excluding non-melanoma skin cancer: 1.15 (95% confidence interval 0.83–1.59). Rate ratios for all malignancies: 0.84 (95% confidence interval 0.62–1.13).
Nationwide cohort, Sweden (Wadström et al., 2017) [23]	Rheumatoid arthritis	National register–based prospective cohort	Tocilizumab n = 1798; abatacept n = 2021; rituximab n = 3586; first tumour necrosis factor inhibitor n = 10,782; second tumour necrosis factor inhibitor n = 4347; vs. conventional therapy n = 46,610 and general population n = 107,491	Not reported as a median; maximum follow-up under 10 years (study period 2006–2015)	Patients with prior malignancy were excluded for each outcome analysed	Crude incidence per 100,000 person-years for first invasive solid or haematologic malignancy: tocilizumab 959, abatacept 1026, rituximab 1074, first tumour necrosis factor inhibitor 978, second tumour necrosis factor inhibitor 917. No statistically significant differences from biologic-naïve patients across 25 drug-outcome comparisons, except an increased risk of squamous cell skin cancer with abatacept.
Danish nationwide registry (Nyboe Andersen et al., 2014) [24]	Inflammatory bowel disease	Nationwide register-based cohort	4553 tumour necrosis factor inhibitor–exposed vs. 51,593 unexposed (56,146 enrolled total)	Median 9.3 years overall (interquartile range 4.2–14.0); median 3.7 years among exposed patients (interquartile range 1.8–6.0)	Not stated	Fully adjusted relative risk of cancer (exposed vs. unexposed): 1.07 (95% confidence interval 0.85–1.36). No significant association by time since first exposure or by cumulative dose.
Brodalumab pharmacovigilance programme (United States) (Lebwohl et al., 2025) [25]	Psoriasis	Post-marketing adverse event surveillance	5449 patients; compared descriptively with PSOLAR (12,095 patients)	7-year surveillance period; no median reported	Not stated	Crude malignancy reporting rate: 1.01 per 100 patients in year 7 (64 malignancies in 55 patients), compared with 1.78 per 100 patients in PSOLAR (excluding non-melanoma skin cancer).
BIOBADASER III (Molina-Collada et al., 2026) [26]	Rheumatoid arthritis	Multicentre national registry (2000–2023)	4635 patients; 187 incident cancers; tumour necrosis factor inhibitor as reference comparator	Median 3.6 years	No—patients with prior malignancy were excluded	Adjusted hazard ratios for cancer excluding non-melanoma skin cancer, vs. tumour necrosis factor inhibitor: interleukin-6 inhibitor 1.2 (95% confidence interval 0.8–1.6); CD20 inhibitor 0.9 (95% confidence interval 0.5–1.4); Janus kinase inhibitor 1.2 (95% confidence interval 0.8–1.8); CTLA4 fusion protein 1.1 (95% confidence interval 0.8–1.6).
Korean national cohort (Song et al., 2025) [27]	Psoriasis and rheumatoid arthritis	Retrospective nationwide cohort	7645 tumour necrosis factor inhibitor user’s vs. 1,520,304 non-users (1,527,949 analysed in total)	Not reported as a median; cumulative exposure assessed over a fixed 2-year window	Excluded	Adjusted hazard ratio for lymphoma in infliximab users: 2.49 (95% confidence interval 1.33–4.66; *p* < 0.01). No significant change in risk for colorectal, liver, lung, kidney, breast, or thyroid cancer (adjusted hazard ratios 0.90–1.20).
REGATE (Morel et al., 2025) [28]	Rheumatoid arthritis	French national prospective cohort; no internal comparator arm	1496 tocilizumab-treated patients	Mean 47 months (standard deviation 15.2); 3990.9 patient-years of exposure; no median reported	Not stated	Incidence of cancer excluding non-melanoma skin cancer: 7.5 per 1000 patient-years. Independent risk factors for solid cancer: older age (hazard ratio 1.05, 95% confidence interval 1.02–1.08), current smoking (hazard ratio 2.65, 95% confidence interval 1.27–5.53), male sex (hazard ratio 2.38, 95% confidence interval 1.18–4.80).
Swedish Rheumatology Quality Register (Huss et al., 2023) [29]	Rheumatoid arthritis and psoriatic arthritis	Prospective cohort linked to the national Cancer Register	10,447 patients with rheumatoid arthritis and 4443 with psoriatic arthritis initiating Janus kinase inhibitors, tumour necrosis factor inhibitors, or other biologics	Median 1.95, 2.83, and 2.49 years for the three rheumatoid arthritis treatment groups, respectively	Not stated	In rheumatoid arthritis, hazard ratio for cancer excluding non-melanoma skin cancer (Janus kinase inhibitor vs. tumour necrosis factor inhibitor): 0.94 (95% confidence interval 0.65–1.38). Hazard ratio for non-melanoma skin cancer: 1.39 (95% confidence interval 1.01–1.91), rising to 2.12 (95% confidence interval 1.15–3.89) at 2 or more years of exposure.
United States population-based cohort (Ma et al., 2025) [30]	Asthma	Population-based cohort, propensity-score matched	14,936 dupilumab-treated vs. 734,126 treated with inhaled corticosteroid/long-acting beta-agonist combination therapy	2018–2024 study period; no median reported	Not stated	Hazard ratio for lymphoma (dupilumab vs. comparator): 1.79 (95% confidence interval 1.19–2.71); for T-cell/natural killer-cell lymphoma specifically: 4.58 (95% confidence interval 1.82–11.53). No significant difference for other malignancies.
Danish nationwide registry (Westermann et al., 2025) [31]	Rheumatoid arthritis	Nationwide register-based cohort	1457 tocilizumab/sarilumab; 1016 abatacept; 690 rituximab; 7458 tumour necrosis factor inhibitor; 11,361 conventional therapy initiations (21,982 total)	Median 2.5, 2.3, 3.5, and 3.7 years for the four biologic groups, and 3.2 years for conventional therapy (interquartile ranges reported in source)	Not stated	No significantly increased hazard ratio for overall cancer with tocilizumab/sarilumab, abatacept, or rituximab (range 0.7–1.1). Abatacept exposure beyond 5 years: hazard ratio 1.41 (95% confidence interval 0.74–2.71) vs. tumour necrosis factor inhibitor. Rituximab and haematologic cancer: hazard ratio 0.09 (95% confidence interval 0.00–2.06) vs. tumour necrosis factor inhibitor.
International TriNetX cohort (Kridin et al., 2024) [32]	Psoriasis	Global population-based cohort, propensity-score matched	15,331 interleukin-17 inhibitor vs. 15,331 tumour necrosis factor inhibitor; 5832 interleukin-23 inhibitor vs. 5832 tumour necrosis factor inhibitor	5 years	Not stated	Interleukin-17 inhibitor vs. tumour necrosis factor inhibitor: hazard ratio for non-Hodgkin lymphoma 0.58 (95% confidence interval 0.40–0.82); colorectal cancer 0.68 (95% confidence interval 0.49–0.95); melanoma 0.52 (95% confidence interval 0.37–0.73). Interleukin-23 inhibitor vs. tumour necrosis factor inhibitor: hazard ratio for non-Hodgkin lymphoma 0.39 (95% confidence interval 0.19–0.78).
Real-world urticaria cohorts (Calzari et al., 2024) [33]	Chronic spontaneous urticaria	Retrospective cohort	296 omalizumab-treated patients; no comparator arm	Median 22.0 months (interquartile range 9.0–45.0)	Not stated	No malignancy signal reported across 8-year cumulative observation.
SUSTAIN Study (Chaparro et al., 2022 [34]	Crohn disease	Retrospective multicentre cohort	463 ustekinumab-treated patients; no comparator arm	Median 15 months	Not stated	50 adverse events in 39 patients (8.4%); one malignancy reported among four severe adverse events.

**Table 3:** Summary of Clinical Trial Data on Biologic-Associated Malignancy Risk.

Trial or Programme	Biologic/Drug Class	Disease Entity	Study Design	Exposed vs. Comparator, n	Median Follow-Up	Prior Malignancy Included	Ke malignancy Findings (Hazard Ratio, Relative Risk, Incidence Rate, 95% Confidence Interval)
VOYAGE 1 and VOYAGE 2 (Blauvelt et al., 2023) [35]	Guselkumab (interleukin-23 inhibitor)	Psoriasis	Pooled long-term extension of two phase 3 randomised trials	1721 guselkumab-treated patients overall; 18 patients with a prior history of malignancy analysed separately; no placebo comparator beyond week 52	Up to 5 years (7166 patient-years overall; mean exposure not separately stated for the prior-malignancy subgroup)	Yes—18 patients with a history of malignancy were specifically analysed	In the overall population, malignancy excluding non-melanoma skin cancer occurred at 0.45 per 100 patient-years and non-melanoma skin cancer at 0.34 per 100 patient-years, consistent with general-population rates. In the prior-malignancy subgroup, one of 18 patients (with reported cases of prostate and breast cancer and non-melanoma skin cancer noted across the wider VOYAGE cohort) experienced a recurrent malignancy.
Pooled analysis of 28 clinical trials (Gottlieb et al., 2022) [36]	Secukinumab (interleukin-17A inhibitor)	Psoriasis, psoriatic arthritis, and ankylosing spondylitis	Integrated phase 2/3 clinical trial and post-marketing surveillance data	12,637 secukinumab-treated patients across indications (10,416.9 psoriasis, 3866.9 psoriatic arthritis, and 1943.1 ankylosing spondylitis patient-years in the trial dataset; no placebo comparator in the pooled long-term analysis)	Up to 5 years (cumulative post-marketing exposure approximately 285,811 patient-years)	Not stated	Standardised incidence ratio for malignancy versus the general population was 0.99 (95% confidence interval 0.82–1.19); the incidence of malignancy was 1 per 100 patient-years or lower across indications.
BE RADIANT open-label extension (Warren et al., 2025) [37]	Bimekizumab (interleukin-17A/F inhibitor)	Psoriasis	Phase 3b randomised trial with double-blind period and open-label extension	691 bimekizumab-exposed patients (including former secukinumab-randomised patients who switched); no placebo comparator in the long-term extension	3 years (1580 patient-years of exposure)	Not stated	Malignancy incidence rate was 1.1 per 100 patient-years (95% confidence interval 0.6–1.8); excluding non-melanoma skin cancer, 0.5 per 100 patient-years (95% confidence interval 0.2–1.0). Rates did not increase with continued exposure.
Integrated phase 2b/3 studies (Mease et al., 2025) [38]	Bimekizumab (interleukin-17A/F inhibitor)	Axial spondyloarthritis and psoriatic arthritis	Pooled safety analysis of six integrated phase 2b/3 studies and open-label extensions	848 patients with axial spondyloarthritis (2034.4 patient-years) and 1407 patients with psoriatic arthritis (2590.8 patient-years); no placebo comparator beyond the initial 16-week period	Median exposure 813 days (axial spondyloarthritis) and 721 days (psoriatic arthritis)	Not stated	Malignancy excluding non-melanoma skin cancer occurred in 5 of 848 patients with axial spondyloarthritis (0.2 per 100 patient-years) and 12 of 1407 patients with psoriatic arthritis (0.5 per 100 patient-years); rates were stable with continued exposure.
Pooled analysis of 25 randomised clinical trials (Merola et al., 2025) [39]	Ixekizumab (interleukin-17A inhibitor)	Psoriasis, psoriatic arthritis, and axial spondyloarthritis	Integrated safety analysis across phase 1–4 trials	6892 patients with psoriasis, 1401 with psoriatic arthritis, and 932 with axial spondyloarthritis; no pooled placebo comparator over the full exposure period	Up to 5 years (psoriasis) and up to 3 years (psoriatic arthritis); cumulative exposure 22,371.1 patient-years	Not stated	Malignant neoplasm incidence rates were 0.8 per 100 patient-years (95% confidence interval 0.7–0.9) in psoriasis, 0.7 per 100 patient-years (95% confidence interval 0.4–1.1) in psoriatic arthritis, and 0.4 per 100 patient-years (95% confidence interval 0.2–0.8) in axial spondyloarthritis. Standardised incidence ratios versus the general United States population (SEER data) were at or below 1 across all three indications, with no increase over successive years of exposure.
LUCENT-3 open-label extension (Sands et al., 2025) [40]	Mirikizumab (interleukin-23 inhibitor)	Ulcerative colitis	Open-label extension of phase 3 induction and maintenance trials	316 patients entered the extension (from 868 induction patients and 365 randomised to maintenance); no placebo comparator in the extension	Up to 152 weeks (approximately 3 years)	Not stated	Malignancy occurred in 0.3% of patients in the safety population, alongside opportunistic infection in 1.8% and hepatic disorders in 3.2%, with no new safety signal identified.
FORTIFY open-label extension (Ferrante et al., 2024) [41]	Risankizumab (interleukin-23 inhibitor)	Crohn’s disease	Open-label extension of a phase 3 maintenance trial	Over 1100 patients entered the open-label extension; no placebo comparator beyond the original 52-week maintenance trial	Up to 152 weeks (approximately 3 years)	Not stated	Rates of adjudicated malignancies remained stable from week 56 to week 152 with no new safety signal identified; two malignancies were reported in total during the extension period.
GALAXI-2 and GALAXI-3 (Panaccione et al., 2025) [42]	Guselkumab (interleukin-23 inhibitor)	Crohn’s disease	Two identically designed phase 3 randomised, placebo- and active comparator-controlled trials	1021 patients in the primary analysis population (1048 randomised and treated) across both trials, with placebo and ustekinumab active comparator arms	48 weeks	Not stated	No increased risk of malignancy was identified during the induction or maintenance phases; the overall safety profile was consistent with that observed in other guselkumab indications.
Pooled analysis of five clinical trials (CLASSIC I and II, CHARM, GAIN, ADHERE) (Osterman et al., 2014) [43]	Adalimumab (tumour necrosis factor inhibitor)	Crohn’s disease	Pooled analysis of randomised controlled trials and a long-term extension study	1594 patients (3050 patient-years of exposure); 44% received concomitant immunomodulator therapy (563 thiopurine, 131 methotrexate); comparator was the general population (Surveillance, Epidemiology, and End Results registry)	Not stated as a median; total pooled exposure 3050 patient-years	Not stated	The incidence of malignancy with adalimumab monotherapy was not greater than that of the general population; co-administration of an immunomodulator with adalimumab was associated with an increased risk of non-melanoma skin cancer and other cancers compared with adalimumab monotherapy.
IM-UNITI long-term extension (Sandborn et al., 2018) [44]	Ustekinumab (interleukin-12/23 inhibitor)	Crohn’s disease	Long-term extension of a phase 3 randomised maintenance trial	298 of 397 randomised patients entered the long-term extension; no placebo comparator beyond the original maintenance trial	Up to 5 years (272 weeks)	Not stated	Six additional malignancies, excluding non-melanoma skin cancer, were reported between weeks 156 and 272 (intraocular melanoma, renal cell carcinoma, endometrial adenocarcinoma, lentigo maligna melanoma, lobular breast carcinoma in situ, and pancreatic carcinoma); no lymphomas were observed throughout the extension.
Pooled safety analysis of six phase 2/3 studies (Ghosh et al., 2024) [45]	Ustekinumab (interleukin-12/23 inhibitor)	Crohn’s disease and ulcerative colitis	Pooled analysis of placebo-controlled induction and maintenance studies and their extensions	2575 ustekinumab-treated patients (4826 patient-years); placebo comparator included within the pooled induction and maintenance periods	Up to 5 years in Crohn’s disease and 4 years in ulcerative colitis	Not stated	Rates of malignancy and major adverse cardiac events were similar between placebo and ustekinumab, or not higher with ustekinumab; malignancies and opportunistic infections, including tuberculosis, were reported infrequently, and no lymphomas were observed.
Integrated safety analysis of five core trials and extensions (Schiff et al., 2011) [46]	Tocilizumab (interleukin-6 receptor inhibitor)	Rheumatoid arthritis	Pooled analysis of five phase 3 trials, two open-label extensions, and one pharmacology study	4009 patients in the all-exposed population (8580 patient-years); 4199 patients in the all-control population from the placebo-controlled portions of the core trials	Mean 2.4 years (median 2.6 years; range 0.0–4.1 years)	Not stated	Malignancy incidence was 1.1 per 100 patient-years in the all-exposed population, comparable to a contemporary United States rheumatoid arthritis cohort rate of 1.3 per 100 patient-years; rates were stable over time with no increase with prolonged exposure.
Pooled analysis of five randomised trials (Simpson et al., 2022) [47]	Tralokinumab (interleukin-13 inhibitor)	Atopic dermatitis	Pooled analysis of one phase 2b and four phase 3 placebo-controlled trials	1605 tralokinumab-treated patients vs. 680 placebo-treated patients in the initial treatment period; 324 patients continued tralokinumab in the maintenance period and 1121 in open-label treatment	Up to 52 weeks in the monotherapy pool; malignancy assessed across the entire trial period	Not stated	Across the entire trial period, malignancy occurred in 0.9% of tralokinumab-treated patients (n = 17; 1.4 per 100 patient-years of exposure) versus 0.7% of placebo-treated patients (n = 5; 2.2 per 100 patient-years), giving a rate ratio of 0.6 (95% confidence interval 0.3–1.5); most events were skin malignancies with no clustering over time.
LIBERTY AD SOLO 1 and SOLO 2 (Thaçi et al., 2019) [48]	Dupilumab (interleukin-4/13 inhibitor)	Atopic dermatitis	Two identically designed phase 3 randomised, placebo-controlled trials	1379 patients randomised to dupilumab or placebo	16 weeks	Not stated	No new safety signal for malignancy was identified during the 16-week treatment period; the safety profile was consistent with previously published results.
Open-label extension across the dupilumab atopic dermatitis programme (Langley et al., 2025) [49]	Dupilumab (interleukin-4/13 inhibitor)	Atopic dermatitis	Integrated analysis of eight phase 3 trials and two open-label extensions	More than 7000 patient-years of cumulative dupilumab exposure across paediatric, adolescent, and adult cohorts	Up to 5 years in the adult open-label extension	Not stated	No new malignancy signal was identified across the integrated long-term safety dataset; serious infections and non-herpetic skin infections were more frequent with placebo than with dupilumab.
MELTEMI and COLUMBA (Bourdin et al., 2024) [50]	Benralizumab and mepolizumab (interleukin-5 pathway inhibitors)	Severe eosinophilic asthma	Two parallel open-label, long-term extension studies	446 patients in MELTEMI (benralizumab every 4 weeks, n = 220; every 8 weeks, n = 226; exposure 784.28 and 797.03 patient-years, respectively) and 347 patients in COLUMBA (mepolizumab; 1201 patient-years)	5 years	Not stated	Malignancies were few and numerically similar between studies; the six malignancies reported in COLUMBA comprised three cases of basal cell carcinoma, two of prostate cancer, and one of breast cancer, with no signal of increased risk from sustained eosinophil depletion.
Pooled analysis of 32 randomised, double-blind, placebo-controlled trials (within a 67-trial programme) (Busse et al., 2012) [51]	Omalizumab (anti-immunoglobulin E)	Asthma	Pooled analysis of phase 1–300 randomised, double-blind, placebo-controlled trials	4254 omalizumab-treated patients vs. 3178 placebo-treated patients	Not stated as a median; trial-level exposure approximately 2978 and 2168 patient-years for omalizumab and placebo, respectively	Not stated	Incidence rates of malignancy were 4.14 per 1000 patient-years (95% confidence interval 2.26–6.94) with omalizumab and 4.45 per 1000 patient-years (95% confidence interval 2.22–7.94) with placebo, giving a rate ratio of 0.93 (95% confidence interval 0.39–2.27); no causal relationship between omalizumab and malignancy was supported.
OCTAVE Open (Sandborn et al., 2022) [52]	Tofacitinib (Janus kinase inhibitor)	Ulcerative colitis	Open-label, long-term extension of the phase 3 OCTAVE programme	944 patients received at least one dose of tofacitinib in OCTAVE Open; no placebo comparator in the extension	Up to 7.0 years	Not stated	Serious infections, malignancies, and venous thromboembolic events were infrequent, with incidence rates consistent with earlier interim analyses of the same programme; no new safety signal emerged with extended treatment duration.
Guselkumab (interleukin-23 inhibitor)	VOYAGE 1 and VOYAGE 2	Psoriasis	Pooled long-term extension of two phase 3 randomised trials	1721 guselkumab-treated patients overall; 18 patients with a prior history of malignancy analysed separately; no placebo comparator beyond week 52	Up to 5 years (7166 patient-years overall; mean exposure not separately stated for the prior-malignancy subgroup)	Yes—18 patients with a history of malignancy were specifically analysed	In the overall population, malignancy excluding non-melanoma skin cancer occurred at 0.45 per 100 patient-years and non-melanoma skin cancer at 0.34 per 100 patient-years, consistent with general-population rates. In the prior-malignancy subgroup, one of 18 patients (with reported cases of prostate and breast cancer and non-melanoma skin cancer noted across the wider VOYAGE cohort) experienced a recurrent malignancy.
Secukinumab (interleukin-17A inhibitor)	Pooled analysis of 28 clinical trials	Psoriasis, psoriatic arthritis, and ankylosing spondylitis	Integrated phase 2/3 clinical trial and post-marketing surveillance data	12,637 secukinumab-treated patients across indications (10,416.9 psoriasis, 3866.9 psoriatic arthritis, and 1943.1 ankylosing spondylitis patient-years in the trial dataset; no placebo comparator in the pooled long-term analysis)	Up to 5 years (cumulative post-marketing exposure approximately 285,811 patient-years)	Not stated	Standardised incidence ratio for malignancy versus the general population was 0.99 (95% confidence interval 0.82–1.19); the incidence of malignancy was 1 per 100 patient-years or lower across indications.
Bimekizumab (interleukin-17A/F inhibitor)	BE RADIANT open-label extension	Psoriasis	Phase 3b randomised trial with double-blind period and open-label extension	691 bimekizumab-exposed patients (including former secukinumab-randomised patients who switched); no placebo comparator in the long-term extension	3 years (1580 patient-years of exposure)	Not stated	Malignancy incidence rate was 1.1 per 100 patient-years (95% confidence interval 0.6–1.8); excluding non-melanoma skin cancer, 0.5 per 100 patient-years (95% confidence interval 0.2–1.0). Rates did not increase with continued exposure.
Bimekizumab (interleukin-17A/F inhibitor)	Integrated phase 2b/3 studies	Axial spondyloarthritis and psoriatic arthritis	Pooled safety analysis of six integrated phase 2b/3 studies and open-label extensions	848 patients with axial spondyloarthritis (2034.4 patient-years) and 1407 patients with psoriatic arthritis (2590.8 patient-years); no placebo comparator beyond the initial 16-week period	Median exposure 813 days (axial spondyloarthritis) and 721 days (psoriatic arthritis)	Not stated	Malignancy excluding non-melanoma skin cancer occurred in 5 of 848 patients with axial spondyloarthritis (0.2 per 100 patient-years) and 12 of 1407 patients with psoriatic arthritis (0.5 per 100 patient-years); rates were stable with continued exposure.
Ixekizumab (interleukin-17A inhibitor)	Pooled analysis of 25 randomised clinical trials	Psoriasis, psoriatic arthritis, and axial spondyloarthritis	Integrated safety analysis across phase 1–4 trials	6892 patients with psoriasis, 1401 with psoriatic arthritis, and 932 with axial spondyloarthritis; no pooled placebo comparator over the full exposure period	Up to 5 years (psoriasis) and up to 3 years (psoriatic arthritis); cumulative exposure 22,371.1 patient-years	Not stated	Malignant neoplasm incidence rates were 0.8 per 100 patient-years (95% confidence interval 0.7–0.9) in psoriasis, 0.7 per 100 patient-years (95% confidence interval 0.4–1.1) in psoriatic arthritis, and 0.4 per 100 patient-years (95% confidence interval 0.2–0.8) in axial spondyloarthritis. Standardised incidence ratios versus the general United States population (SEER data) were at or below 1 across all three indications, with no increase over successive years of exposure.
Mirikizumab (interleukin-23 inhibitor)	LUCENT-3 open-label extension	Ulcerative colitis	Open-label extension of phase 3 induction and maintenance trials	316 patients entered the extension (from 868 induction patients and 365 randomised to maintenance); no placebo comparator in the extension	Up to 152 weeks (approximately 3 years)	Not stated	Malignancy occurred in 0.3% of patients in the safety population, alongside opportunistic infection in 1.8% and hepatic disorders in 3.2%, with no new safety signal identified.
Risankizumab (interleukin-23 inhibitor)	FORTIFY open-label extension	Crohn’s disease	Open-label extension of a phase 3 maintenance trial	Over 1100 patients entered the open-label extension; no placebo comparator beyond the original 52-week maintenance trial	Up to 152 weeks (approximately 3 years)	Not stated	Rates of adjudicated malignancies remained stable from week 56 to week 152 with no new safety signal identified; two malignancies were reported in total during the extension period.
Guselkumab (interleukin-23 inhibitor)	GALAXI-2 and GALAXI-3	Crohn’s disease	Two identically designed phase 3 randomised, placebo- and active comparator-controlled trials	1021 patients in the primary analysis population (1048 randomised and treated) across both trials, with placebo and ustekinumab active comparator arms	48 weeks	Not stated	No increased risk of malignancy was identified during the induction or maintenance phases; the overall safety profile was consistent with that observed in other guselkumab indications.
Adalimumab (tumour necrosis factor inhibitor)	Pooled analysis of five clinical trials (CLASSIC I and II, CHARM, GAIN, ADHERE)	Crohn’s disease	Pooled analysis of randomised controlled trials and a long-term extension study	1594 patients (3050 patient-years of exposure); 44% received concomitant immunomodulator therapy (563 thiopurine, 131 methotrexate); comparator was the general population (Surveillance, Epidemiology, and End Results registry)	Not stated as a median; total pooled exposure 3050 patient-years	Not stated	The incidence of malignancy with adalimumab monotherapy was not greater than that of the general population; co-administration of an immunomodulator with adalimumab was associated with an increased risk of non-melanoma skin cancer and other cancers compared with adalimumab monotherapy.
Ustekinumab (interleukin-12/23 inhibitor)	IM-UNITI long-term extension	Crohn’s disease	Long-term extension of a phase 3 randomised maintenance trial	298 of 397 randomised patients entered the long-term extension; no placebo comparator beyond the original maintenance trial	Up to 5 years (272 weeks)	Not stated	Six additional malignancies, excluding non-melanoma skin cancer, were reported between weeks 156 and 272 (intraocular melanoma, renal cell carcinoma, endometrial adenocarcinoma, lentigo maligna melanoma, lobular breast carcinoma in situ, and pancreatic carcinoma); no lymphomas were observed throughout the extension.
Ustekinumab (interleukin-12/23 inhibitor)	Pooled safety analysis of six phase 2/3 studies	Crohn’s disease and ulcerative colitis	Pooled analysis of placebo-controlled induction and maintenance studies and their extensions	2575 ustekinumab-treated patients (4826 patient-years); placebo comparator included within the pooled induction and maintenance periods	Up to 5 years in Crohn’s disease and 4 years in ulcerative colitis	Not stated	Rates of malignancy and major adverse cardiac events were similar between placebo and ustekinumab, or not higher with ustekinumab; malignancies and opportunistic infections, including tuberculosis, were reported infrequently, and no lymphomas were observed.
Tocilizumab (interleukin-6 receptor inhibitor)	Integrated safety analysis of five core trials and extensions	Rheumatoid arthritis	Pooled analysis of five phase 3 trials, two open-label extensions, and one pharmacology study	4009 patients in the all-exposed population (8580 patient-years); 4199 patients in the all-control population from the placebo-controlled portions of the core trials	Mean 2.4 years (median 2.6 years; range 0.0–4.1 years)	Not stated	Malignancy incidence was 1.1 per 100 patient-years in the all-exposed population, comparable to a contemporary United States rheumatoid arthritis cohort rate of 1.3 per 100 patient-years; rates were stable over time with no increase with prolonged exposure.
Tralokinumab (interleukin-13 inhibitor)	Pooled analysis of five randomised trials	Atopic dermatitis	Pooled analysis of one phase 2b and four phase 3 placebo-controlled trials	1605 tralokinumab-treated patients vs. 680 placebo-treated patients in the initial treatment period; 324 patients continued tralokinumab in the maintenance period and 1121 in open-label treatment	Up to 52 weeks in the monotherapy pool; malignancy assessed across the entire trial period	Not stated	Across the entire trial period, malignancy occurred in 0.9% of tralokinumab-treated patients (n = 17; 1.4 per 100 patient-years of exposure) versus 0.7% of placebo-treated patients (n = 5; 2.2 per 100 patient-years), giving a rate ratio of 0.6 (95% confidence interval 0.3–1.5); most events were skin malignancies with no clustering over time.
Dupilumab (interleukin-4/13 inhibitor)	LIBERTY AD SOLO 1 and SOLO 2	Atopic dermatitis	Two identically designed phase 3 randomised, placebo-controlled trials	1379 patients randomised to dupilumab or placebo	16 weeks	Not stated	No new safety signal for malignancy was identified during the 16-week treatment period; the safety profile was consistent with previously published results.
Dupilumab (interleukin-4/13 inhibitor)	Open-label extension across the dupilumab atopic dermatitis programme	Atopic dermatitis	Integrated analysis of eight phase 3 trials and two open-label extensions	More than 7000 patient-years of cumulative dupilumab exposure across paediatric, adolescent, and adult cohorts	Up to 5 years in the adult open-label extension	Not stated	No new malignancy signal was identified across the integrated long-term safety dataset; serious infections and non-herpetic skin infections were more frequent with placebo than with dupilumab.
Benralizumab and mepolizumab (interleukin-5 pathway inhibitors)	MELTEMI and COLUMBA	Severe eosinophilic asthma	Two parallel open-label, long-term extension studies	446 patients in MELTEMI (benralizumab every 4 weeks, n = 220; every 8 weeks, n = 226; exposure 784.28 and 797.03 patient-years, respectively) and 347 patients in COLUMBA (mepolizumab; 1201 patient-years)	5 years	Not stated	Malignancies were few and numerically similar between studies; the six malignancies reported in COLUMBA comprised three cases of basal cell carcinoma, two of prostate cancer, and one of breast cancer, with no signal of increased risk from sustained eosinophil depletion.
Omalizumab (anti-immunoglobulin E)	Pooled analysis of 32 randomised, double-blind, placebo-controlled trials (within a 67-trial programme)	Asthma	Pooled analysis of phase 1–300 randomised, double-blind, placebo-controlled trials	4254 omalizumab-treated patients vs. 3178 placebo-treated patients	Not stated as a median; trial-level exposure approximately 2978 and 2168 patient-years for omalizumab and placebo, respectively	Not stated	Incidence rates of malignancy were 4.14 per 1000 patient-years (95% confidence interval 2.26–6.94) with omalizumab and 4.45 per 1000 patient-years (95% confidence interval 2.22–7.94) with placebo, giving a rate ratio of 0.93 (95% confidence interval 0.39–2.27); no causal relationship between omalizumab and malignancy was supported.
Tofacitinib (Janus kinase inhibitor)	OCTAVE Open	Ulcerative colitis	Open-label, long-term extension of the phase 3 OCTAVE programme	944 patients received at least one dose of tofacitinib in OCTAVE Open; no placebo comparator in the extension	Up to 7.0 years	Not stated	Serious infections, malignancies, and venous thromboembolic events were infrequent, with incidence rates consistent with earlier interim analyses of the same programme; no new safety signal emerged with extended treatment duration.
Guselkumab (interleukin-23 inhibitor)	VOYAGE 1 and VOYAGE 2	Psoriasis	Pooled long-term extension of two phase 3 randomised trials	1721 guselkumab-treated patients overall; 18 patients with a prior history of malignancy analysed separately; no placebo comparator beyond week 52	Up to 5 years (7166 patient-years overall; mean exposure not separately stated for the prior-malignancy subgroup)	Yes—18 patients with a history of malignancy were specifically analysed	In the overall population, malignancy excluding non-melanoma skin cancer occurred at 0.45 per 100 patient-years and non-melanoma skin cancer at 0.34 per 100 patient-years, consistent with general-population rates. In the prior-malignancy subgroup, one of 18 patients (with reported cases of prostate and breast cancer and non-melanoma skin cancer noted across the wider VOYAGE cohort) experienced a recurrent malignancy.
Secukinumab (interleukin-17A inhibitor)	Pooled analysis of 28 clinical trials	Psoriasis, psoriatic arthritis, and ankylosing spondylitis	Integrated phase 2/3 clinical trial and post-marketing surveillance data	12,637 secukinumab-treated patients across indications (10,416.9 psoriasis, 3866.9 psoriatic arthritis, and 1943.1 ankylosing spondylitis patient-years in the trial dataset; no placebo comparator in the pooled long-term analysis)	Up to 5 years (cumulative post-marketing exposure approximately 285,811 patient-years)	Not stated	Standardised incidence ratio for malignancy versus the general population was 0.99 (95% confidence interval 0.82–1.19); the incidence of malignancy was 1 per 100 patient-years or lower across indications.
Bimekizumab (interleukin-17A/F inhibitor)	BE RADIANT open-label extension	Psoriasis	Phase 3b randomised trial with double-blind period and open-label extension	691 bimekizumab-exposed patients (including former secukinumab-randomised patients who switched); no placebo comparator in the long-term extension	3 years (1580 patient-years of exposure)	Not stated	Malignancy incidence rate was 1.1 per 100 patient-years (95% confidence interval 0.6–1.8); excluding non-melanoma skin cancer, 0.5 per 100 patient-years (95% confidence interval 0.2–1.0). Rates did not increase with continued exposure.
Bimekizumab (interleukin-17A/F inhibitor)	Integrated phase 2b/3 studies	Axial spondyloarthritis and psoriatic arthritis	Pooled safety analysis of six integrated phase 2b/3 studies and open-label extensions	848 patients with axial spondyloarthritis (2034.4 patient-years) and 1407 patients with psoriatic arthritis (2590.8 patient-years); no placebo comparator beyond the initial 16-week period	Median exposure 813 days (axial spondyloarthritis) and 721 days (psoriatic arthritis)	Not stated	Malignancy excluding non-melanoma skin cancer occurred in 5 of 848 patients with axial spondyloarthritis (0.2 per 100 patient-years) and 12 of 1407 patients with psoriatic arthritis (0.5 per 100 patient-years); rates were stable with continued exposure.
Ixekizumab (interleukin-17A inhibitor)	Pooled analysis of 25 randomised clinical trials	Psoriasis, psoriatic arthritis, and axial spondyloarthritis	Integrated safety analysis across phase 1–4 trials	6892 patients with psoriasis, 1401 with psoriatic arthritis, and 932 with axial spondyloarthritis; no pooled placebo comparator over the full exposure period	Up to 5 years (psoriasis) and up to 3 years (psoriatic arthritis); cumulative exposure 22,371.1 patient-years	Not stated	Malignant neoplasm incidence rates were 0.8 per 100 patient-years (95% confidence interval 0.7–0.9) in psoriasis, 0.7 per 100 patient-years (95% confidence interval 0.4–1.1) in psoriatic arthritis, and 0.4 per 100 patient-years (95% confidence interval 0.2–0.8) in axial spondyloarthritis. Standardised incidence ratios versus the general United States population (SEER data) were at or below 1 across all three indications, with no increase over successive years of exposure.
Mirikizumab (interleukin-23 inhibitor)	LUCENT-3 open-label extension	Ulcerative colitis	Open-label extension of phase 3 induction and maintenance trials	316 patients entered the extension (from 868 induction patients and 365 randomised to maintenance); no placebo comparator in the extension	Up to 152 weeks (approximately 3 years)	Not stated	Malignancy occurred in 0.3% of patients in the safety population, alongside opportunistic infection in 1.8% and hepatic disorders in 3.2%, with no new safety signal identified.
Risankizumab (interleukin-23 inhibitor)	FORTIFY open-label extension	Crohn’s disease	Open-label extension of a phase 3 maintenance trial	Over 1100 patients entered the open-label extension; no placebo comparator beyond the original 52-week maintenance trial	Up to 152 weeks (approximately 3 years)	Not stated	Rates of adjudicated malignancies remained stable from week 56 to week 152 with no new safety signal identified; two malignancies were reported in total during the extension period.
Guselkumab (interleukin-23 inhibitor)	GALAXI-2 and GALAXI-3	Crohn’s disease	Two identically designed phase 3 randomised, placebo- and active comparator-controlled trials	1021 patients in the primary analysis population (1048 randomised and treated) across both trials, with placebo and ustekinumab active comparator arms	48 weeks	Not stated	No increased risk of malignancy was identified during the induction or maintenance phases; the overall safety profile was consistent with that observed in other guselkumab indications.
Adalimumab (tumour necrosis factor inhibitor)	Pooled analysis of five clinical trials (CLASSIC I and II, CHARM, GAIN, ADHERE)	Crohn’s disease	Pooled analysis of randomised controlled trials and a long-term extension study	1594 patients (3050 patient-years of exposure); 44% received concomitant immunomodulator therapy (563 thiopurine, 131 methotrexate); comparator was the general population (Surveillance, Epidemiology, and End Results registry)	Not stated as a median; total pooled exposure 3050 patient-years	Not stated	The incidence of malignancy with adalimumab monotherapy was not greater than that of the general population; co-administration of an immunomodulator with adalimumab was associated with an increased risk of non-melanoma skin cancer and other cancers compared with adalimumab monotherapy.
Ustekinumab (interleukin-12/23 inhibitor)	IM-UNITI long-term extension	Crohn’s disease	Long-term extension of a phase 3 randomised maintenance trial	298 of 397 randomised patients entered the long-term extension; no placebo comparator beyond the original maintenance trial	Up to 5 years (272 weeks)	Not stated	Six additional malignancies, excluding non-melanoma skin cancer, were reported between weeks 156 and 272 (intraocular melanoma, renal cell carcinoma, endometrial adenocarcinoma, lentigo maligna melanoma, lobular breast carcinoma in situ, and pancreatic carcinoma); no lymphomas were observed throughout the extension.
Ustekinumab (interleukin-12/23 inhibitor)	Pooled safety analysis of six phase 2/3 studies	Crohn’s disease and ulcerative colitis	Pooled analysis of placebo-controlled induction and maintenance studies and their extensions	2575 ustekinumab-treated patients (4826 patient-years); placebo comparator included within the pooled induction and maintenance periods	Up to 5 years in Crohn’s disease and 4 years in ulcerative colitis	Not stated	Rates of malignancy and major adverse cardiac events were similar between placebo and ustekinumab, or not higher with ustekinumab; malignancies and opportunistic infections, including tuberculosis, were reported infrequently, and no lymphomas were observed.
Tocilizumab (interleukin-6 receptor inhibitor)	Integrated safety analysis of five core trials and extensions	Rheumatoid arthritis	Pooled analysis of five phase 3 trials, two open-label extensions, and one pharmacology study	4009 patients in the all-exposed population (8580 patient-years); 4199 patients in the all-control population from the placebo-controlled portions of the core trials	Mean 2.4 years (median 2.6 years; range 0.0–4.1 years)	Not stated	Malignancy incidence was 1.1 per 100 patient-years in the all-exposed population, comparable to a contemporary United States rheumatoid arthritis cohort rate of 1.3 per 100 patient-years; rates were stable over time with no increase with prolonged exposure.
Tralokinumab (interleukin-13 inhibitor)	Pooled analysis of five randomised trials	Atopic dermatitis	Pooled analysis of one phase 2b and four phase 3 placebo-controlled trials	1605 tralokinumab-treated patients vs. 680 placebo-treated patients in the initial treatment period; 324 patients continued tralokinumab in the maintenance period and 1121 in open-label treatment	Up to 52 weeks in the monotherapy pool; malignancy assessed across the entire trial period	Not stated	Across the entire trial period, malignancy occurred in 0.9% of tralokinumab-treated patients (n = 17; 1.4 per 100 patient-years of exposure) versus 0.7% of placebo-treated patients (n = 5; 2.2 per 100 patient-years), giving a rate ratio of 0.6 (95% confidence interval 0.3–1.5); most events were skin malignancies with no clustering over time.
Dupilumab (interleukin-4/13 inhibitor)	LIBERTY AD SOLO 1 and SOLO 2	Atopic dermatitis	Two identically designed phase 3 randomised, placebo-controlled trials	1379 patients randomised to dupilumab or placebo	16 weeks	Not stated	No new safety signal for malignancy was identified during the 16-week treatment period; the safety profile was consistent with previously published results.
Dupilumab (interleukin-4/13 inhibitor)	Open-label extension across the dupilumab atopic dermatitis programme	Atopic dermatitis	Integrated analysis of eight phase 3 trials and two open-label extensions	More than 7000 patient-years of cumulative dupilumab exposure across paediatric, adolescent, and adult cohorts	Up to 5 years in the adult open-label extension	Not stated	No new malignancy signal was identified across the integrated long-term safety dataset; serious infections and non-herpetic skin infections were more frequent with placebo than with dupilumab.
Benralizumab and mepolizumab (interleukin-5 pathway inhibitors)	MELTEMI and COLUMBA	Severe eosinophilic asthma	Two parallel open-label, long-term extension studies	446 patients in MELTEMI (benralizumab every 4 weeks, n = 220; every 8 weeks, n = 226; exposure 784.28 and 797.03 patient-years, respectively) and 347 patients in COLUMBA (mepolizumab; 1201 patient-years)	5 years	Not stated	Malignancies were few and numerically similar between studies; the six malignancies reported in COLUMBA comprised three cases of basal cell carcinoma, two of prostate cancer, and one of breast cancer, with no signal of increased risk from sustained eosinophil depletion.
Omalizumab (anti-immunoglobulin E)	Pooled analysis of 32 randomised, double-blind, placebo-controlled trials (within a 67-trial programme)	Asthma	Pooled analysis of phase 1–300 randomised, double-blind, placebo-controlled trials	4254 omalizumab-treated patients vs. 3178 placebo-treated patients	Not stated as a median; trial-level exposure approximately 2978 and 2168 patient-years for omalizumab and placebo, respectively	Not stated	Incidence rates of malignancy were 4.14 per 1000 patient-years (95% confidence interval 2.26–6.94) with omalizumab and 4.45 per 1000 patient-years (95% confidence interval 2.22–7.94) with placebo, giving a rate ratio of 0.93 (95% confidence interval 0.39–2.27); no causal relationship between omalizumab and malignancy was supported.
Tofacitinib (Janus kinase inhibitor)	OCTAVE Open	Ulcerative colitis	Open-label, long-term extension of the phase 3 OCTAVE programme	944 patients received at least one dose of tofacitinib in OCTAVE Open; no placebo comparator in the extension	Up to 7.0 years	Not stated	Serious infections, malignancies, and venous thromboembolic events were infrequent, with incidence rates consistent with earlier interim analyses of the same programme; no new safety signal emerged with extended treatment duration.

## 2. Malignancies in Patients with Auto-Immune Disease

### 2.1. Psoriasis

Patients with psoriasis are purported to be at increased risk of malignancy [1]. The biological plausibility of this association is supported by the role of chronic inflammation in psoriasis and its contribution to carcinogenesis [1,2]. The most consistently reported associations are with immunosensitive malignancies, including lymphoma and skin cancers [3,4,5,6,7,8]. Other reported site-specific cancers include those of the oral cavity, larynx, pharynx, colon, lung, and kidney [3,8,9,10,11,53]. However, a 2015 systematic review found no increased overall malignancy risk across all cancers, lymphoma, melanoma, prostate, colorectal, and breast cancer, other than nonmelanomatous skin cancer [54]. Observed excess risks in other studies [3,4,5,6,7,8] may reflect treatment exposures, lifestyle factors, or surveillance bias.

The first meta-analysis by Trafford et al. evaluating cancer mortality in psoriasis demonstrated substantial attenuation of risk after adjustment for smoking, alcohol use, and obesity [1]. However, Pouplard et al. reported increased risks of selected solid tumours (respiratory tract, upper aerodigestive tract, urinary tract, and liver cancers), haematological malignancies (non-Hodgkin lymphoma), and skin cancers (squamous cell carcinoma and basal cell carcinoma) [55]. Smoking prevalence is high among patients with psoriasis [56], and certain site-specific cancers, including oesophageal and liver cancer, are independently associated with obesity, smoking, and alcohol consumption [57].

Older systemic therapies, including PUVA, cyclosporine, and possibly methotrexate, have also been associated with increased malignancy risk [58,59,60]. Adjustment for lifestyle factors may be methodologically complex, as these variables may lie on the causal pathway. Notably, few studies report duration of treatment exposure, underscoring the need for prospective pharmacovigilance registries.

Overall, observational evidence supports an association between psoriasis and selected malignancies, but whether psoriasis independently increases malignancy risk remains uncertain when treatment exposure, lifestyle confounding, and surveillance bias are considered [1].

### 2.2. Systemic Rheumatic Disease

Rheumatic diseases and malignancy have a complex bidirectional association. Dermatomyositis, polymyositis, rheumatoid arthritis, systemic lupus erythematosus, Sjögren’s syndrome, and systemic sclerosis are linked to increased cancer risk in and of themselves and may occasionally represent paraneoplastic autoimmunity. Furthermore, immunosuppressive therapies used in these conditions can contribute to malignancy risk [61]. Malignancies can initially manifest with musculoskeletal symptoms affecting joints, muscles, or peri-articular tissues. Hematologic cancers, particularly leukaemia and lymphomas, are most frequently implicated, although solid tumours may also give rise to paraneoplastic rheumatic features [62]. Additionally, oncologic treatments can precipitate immune-mediated rheumatic syndromes.

Across multiple meta-analyses and large cohort studies, systemic rheumatic diseases are consistently associated with a modest-to-substantial increase in overall malignancy risk, although the magnitude and tumour type vary by condition [61,63]. Rheumatoid arthritis and systemic lupus erythematosus confer a small but significant elevation in overall cancer risk with a predominance of lymphoproliferative malignancies and lung cancer [64,65]. Systemic sclerosis is associated with a higher relative risk, particularly for lung and selected solid organ cancers [66]. The strongest associations are observed in idiopathic inflammatory myopathies, especially dermatomyositis, where overall cancer risk is markedly elevated, with increased incidence across a broad range of solid tumours [67]. Extended myositis antibody panels further help the risk of malignancy stratification with Anti-TIF1-γ and anti-NXP2 having the strongest associations. Sjögren’s syndrome and ANCA-associated vasculitis have also demonstrated a higher overall malignancy risk, notably driven by non-Hodgkin lymphoma and selected solid tumours [68].

Collectively, these data highlight important disease-specific patterns of cancer susceptibility that have implications for risk stratification and malignancy surveillance in rheumatology practice.

### 2.3. Inflammatory Bowel Disease

The risk of colorectal cancer is increased in patients with IBD compared to the general population. A population-based cohort study of over 96,000 patients with ulcerative colitis found that compared with those without UC, those with UC are at increased risk of developing colorectal cancer, are often diagnosed with less advanced colorectal cancers, and are at increased risk of dying from it [69]. For patients with UC, the duration, extent and activity of disease affects the likelihood of colorectal cancer developing [69,70,71,72].

Similarly for patients with Crohn’s disease (CD), colon cancer risks are similar to those seen in UC [73,74]. A 1990 population-based study found the relative risk of colon cancer was 2.5 in CD overall, but if their disease was limited to the colon, the relative risk increased to 5.6 [73,75]. Other malignancies reported in patients with CD include cancers of the liver, pancreas, lung, prostate, testis, and kidney, (squamous cell) skin cancers, endocrine tumours, and leukaemia [76]. The strength of these associations is unclear; however, one of the most consistent findings in other studies has been an association with haematologic malignancies [76,77].

### 2.4. Chronic Airway Inflammatory Disease

Chronic airway inflammatory disorders such as asthma and Chronic obstructive pulmonary disease (COPD), although not prototypical immune-mediated inflammatory diseases, provide a relevant comparator for understanding malignancy risk in conditions characterised by chronic immune dysregulation and increasingly treated with biologic or targeted therapies.

Across large population-based studies, asthma is associated with a modest overall increased risk of malignancy, with reported hazard ratios for incident malignancy ranging from 1.19 to 1.44 across diverse populations and healthcare systems [78,79,80,81,82].

The most consistent and biologically plausible signal relates to lung cancer. Multiple cohort studies, including the HUNT study, demonstrate a higher lung cancer incidence among adults with active or partially controlled asthma compared with those without asthma, even after adjustment for potential confounders [83]. This association is observed in both smokers and never-smokers, although effect estimates are higher in current or former smokers [80,82,84]. Recent Mendelian randomisation analyses further support a potential causal association between genetic predisposition to asthma and lung cancer [84]. Associations with breast and stomach cancer have also been reported previously [78,85], potentially reflecting chronic inflammation pathways involved in asthma [86]. However, findings across studies are heterogeneous, and some studies report no association between asthma and overall cancer risk [87,88]. Further research is required to clarify underlying mechanisms and to better define risk estimates for non-lung cancers [79,89].

Similar observations have been made in COPD, where chronic airway inflammation confers an increased lung cancer risk independent of smoking, reinforcing the broader concept that persistent airway immune activation contributes to carcinogenesis [90].

## 3. Biologic and Targeted Therapies in the Context of Malignancy

Immunomodulatory agents and biologic therapies are increasingly used by many medical specialties for immune-mediated disease; they are targeted therapies which downregulate these overactive immune pathways, delivering improved outcomes, but immunologic effects can be of concern in the setting of malignancy. Oncologists are often consulted when patients with a past or current malignancy require these systemic treatments. This complex patient cohort requires careful consideration regarding the commencement or cessation of newer biologic treatments.

Generally, immunosuppressants may contribute to the development of new cancers and the recurrence of existing ones through several mechanisms, including reduced immunosurveillance [91], enhanced activity of oncogenic viruses [92], and direct DNA alteration [93]. These mechanisms vary significantly across different immunosuppressants and cancer types. The interplay between the chosen biologic agent, the associated incident risk in patients with no cancer history, recurrence risk in patients with prior malignancy, promotion risk in patients with active cancer, and the impact on their quality of life are all essential factors which require considered deliberation. Direct, high-level evidence to address these concerns is often lacking. This review aims to explore the interface between medical specialties and oncologists for those patients with a chronic medical diagnosis requiring treatment with a biologic agent and a history of malignancy.

### 3.1. Drug Class Evidence

The available evidence for malignancy risk differs across drug classes and diseases and is most robust where long-running registries and real-world cohorts exist. In contrast, randomised trials often have short follow-up and frequently exclude patients with recent malignancy, limiting their ability to quantify malignancy incidence or recurrence risk [35].

#### 3.1.1. TNF-Inhibitors in Dermatology

Tumour necrosis factor (TNF) is a cytokine which exerts necrotising effects when exposed to tumour cells in vitro and is produced by activated T cells and macrophages. TNF is now known to have pro- and antitumorigenic effects which are dependent on unique conditions, the type of producer and responder cell, the type of TNF receptor involved, and the tissue environment. TNF-inhibitors are a widely used treatment for inflammatory dermatoses, with most data being from those patients with psoriasis.

As previously discussed, patients with psoriasis may have an increased risk of incident malignancy, particularly nonmelanoma skin cancers and lymphomas [94]. A 2020 systematic review and meta-analysis of 112 studies and over 2 million patients found that the overall prevalence of cancer in patients with psoriasis was 4.78%. The authors noted no increased risk of incident cancer in those patients treated with a biologic agent; however, they did not include details on the type of biologic agent [94].

The dermatology literature indicates that TNF-inhibitors have been associated with increased risk of lymphoma and nonmelanomatous skin cancers [95,96,97]. A retrospective cohort study of Korean patients with RA or psoriasis taking TNF-inhibitors found no increase in incident cancer but did find an increased risk of lymphoma and leukaemia, particularly for infliximab [27].

The Psoriasis Longitudinal Assessment and Registry (PSOLAR), which is a US-based registry of over 12,000 patients, found that TNF-inhibitor treatment for over 12 months increased the risk of incident lung cancer. However, the confidence intervals in the report were wide and the number of events were low [95]. Further research from the PSOLAR indicated no statistically significant risk of incident malignancy when individual anti-TNF agents were considered [12]. A systematic review of psoriasis patients completed in 2018 found no increase in incident malignancy, except for nonmelanomatous skin cancers, with TNF-inhibitors when compared to the U.S. population [54]. The ESPRIT registry similarly showed no significant increased cancer risk in patients with psoriasis treated with TNF-inhibitors over a 7-year period [18].

Cross-specialty evidence including phase II trials demonstrating the efficacy of infliximab in metastatic renal cell cancer show the potential for underutilisation of these medications because of safety concerns and poor dissemination of relevant data [98]. An umbrella review of potential cancer risk associated with common medications, including TNF-inhibitors, noted the pooled estimate in a larger scale meta-analysis did not demonstrate an increased risk of incident malignancy (RR 0.95) [99].

Overall, available dermatology registry and synthesis data do not demonstrate a consistent increase in overall incident malignancy risk with TNF-inhibitors in psoriasis, with the most consistent signals relating to nonmelanomatous skin cancer and selected site-specific outcomes [12,18,54,95,96]. The evidence base for patients with a current or historical diagnosis of malignancy is lacking. There have been no prospective or randomised studies where patients with psoriasis and a history of malignancy are treated with an anti-TNF agent.

#### 3.1.2. TNF-Inhibitors in Gastroenterology

TNF-inhibitors have a pivotal role in the management of IBD, which includes CD and UC. TNF-inhibitor data for IBD patients should be interpreted in the context of co-prescription with thiopurines. It has been established that receiving thiopurines carries an increased risk of lymphoproliferative disorders, particularly when prescribed in addition to TNF-inhibitors [100,101]. A meta-analysis in 2008 found no increased incidence of malignancy with TNF-inhibitors, though patients with a history of malignancy were not included in the studies [102].

The most relevant data for the prior malignancy cohort of IBD patients are from Axelrad et al., who performed a retrospective analysis of 333 individuals with IBD with a history of cancer and noted no associated increased risk in incident cancer compared with patients who had not received a TNF-inhibitor or antimetabolite [103]. All other data are relevant to incident malignancy, such as a 2014 systematic review which did not find an increased risk of malignancy in patients with IBD, but had the caveats of excluding patients with a history of malignancy and a maximum follow-up duration of 1 year [104]. Equally, a pooled analysis of clinical trials for adalimumab found no increased incident cancer risk, unless it was co-prescribed with another immunomodulatory, providing reassuring evidence for its use as a monotherapy [43].

In the TREAT Registry study, the minimum follow-up period for active patients was approximately 5 years. The authors found no association of systemic therapy and malignancy [17]. An additional large Danish study found no increased overall cancer risk in IBD patients treated with TNF-alpha inhibitors after a median follow-up of 3.7 years. Current evidence indicates that TNF blockade alone does not substantially elevate long-term cancer risk, even with follow-up extending to 19 years [24]. Large cohort and synthesis studies continue to show no clear increase in overall incident cancer with anti-TNF monotherapy in IBD, and the most consistent signals relate to nonmelanomatous skin cancer and potentially melanoma [105]. A recent retrospective review observed a low prevalence rate of cancer, even in the context of a wide variation in biologic and immunosuppressive treatments being used [106]. In patients with pre-existing malignancy, the most directly applicable evidence is observational, and available IBD data do not show an excess of new cancers among those exposed to anti-TNF agents [103].

#### 3.1.3. TNF-Inhibitors in Rheumatology

Rheumatic diseases and cancer share complex bidirectional associations. Disorders such as dermatomyositis, rheumatoid arthritis (RA) and systemic lupus erythematosus (SLE) carry an elevated malignancy risk and, in some cases, may arise secondary to cancer-induced autoimmunity [107]. There is a paucity of data regarding cancer risk in patients with psoriatic arthritis (PSA) [94], but it is generally accepted that inflammatory arthropathies are associated with malignancy in and of themselves [108].

TNF-inhibitors are an essential biologic agent for the treatment of rheumatoid arthritis, psoriatic arthritis and spondyloarthropathies. The German biologics register (RABBIT) was set up to investigate malignancies in patients with RA receiving systemic therapy. This registry reported no significant difference in either the overall incidence of malignancies or recurrent malignancies in patients exposed to TNF-inhibitors or anakinra [14]. The British Society for Rheumatology (BSR) Biologics Register, a prospective observational study established in 2001, identified 293 patients with a prior history of malignancy treated with TNF-inhibitors and found no increased risk in incident malignancy when compared with those receiving DMARD therapy [13]. Additionally, a systematic review for the 2019 update of the EULAR recommendations for the management of psoriatic arthritis did not reveal new malignancy signals for TNF-inhibitors [109].

A large cohort of RA patients from the Swedish cancer registry showed lymphoma risk was not elevated in anti-TNF treated patients when compared to other patient groups [110]. A 2006 [111] and 2011 [112] systematic review and meta-analysis found TNF-inhibitors do not increase the risk of malignancy, particularly lymphoma. The 2006 review did note a significant increase in malignancy rate with higher doses of TNF-inhibitors [111]. A further study explored the incident cancer cases in over 13,000 patients from 1998 to 2005 (49,000 patient years) and found a positive association between biologic therapy and skin cancer but no association with biologic therapy and other malignancies. The biologics included infliximab, etanercept, adalimumab and anakinra [113]. Similarly, data from Swedish (Anti-Rheumatic Therapy in Sweden (ARTIS)) and Danish (DANBIO) biologics registers, which included 8703 patients, found in patients with spondyloarthropathies that TNF-inhibitors were not associated with increased risks of malignancy [16]. This was noted in previous research from the DANBIO registry in 2013 for patients with RA [114]. A more recent systematic review published in 2025 similarly found no increased risk of malignancy with TNF-inhibitors, though the median length of follow-up was 41 weeks (interquartile range of 24–64 weeks) [115].

A randomised non-inferiority trial comparing tofacitinib with anti-TNF therapy (median follow-up 4 years) reported an incident cancer rate of 2.9% (42/1451 patients) in the tofacitinib group—statistically higher than the rate in the anti-TNF group, though the absence of a conventional DMARD arm limits broader comparisons [116]. A Danish nationwide registry-based cohort study of RA patients assessed cancer prevalence in several groups of patients taking systemic agents and found the TNF-inhibitor and bDMARD-naïve groups to be statistically comparable to each other and to other RA patients taking tocilizumab/sarilumab, abatacept or rituximab [31].

Regarding cancer recurrence in patients with a history of malignancy, Westermann et al. also reviewed the cancer recurrence risk with bDMARD treatment in patients with RA and a history of cancer (breast, colorectal, melanoma, bladder, endometrial and lung) and found no statistically significant increased hazard ratio for cancer recurrence for any type of DMARD, TNF-inhibitor or rituximab [117]. However, the majority of data related to incident malignancy, such as the BIOBADASER III registry data for RA patients from 2000 to 2023, found no increased cancer risk associated with a biologic agent or targeted synthetic DMARD when compared to TNF-inhibitors, though patients with a history of malignancy were excluded [26]. Similarly, the Swedish Rheumatology Quality Register (2016–2020 prospective data) found reassuring hazard ratios for malignancy in patients receiving TNF-inhibitors, though follow-up time was relatively shorter at less than 3 years [29].

The most recent EULAR guidelines recommend TNF-inhibitors in patients with a history of solid organ malignancy, as it has the largest body of evidence indicating no significant association with malignancy [108]. Across rheumatology registries, TNF-inhibitors have the largest evidence base in patients with prior malignancy, and recurrence signals have not been consistently demonstrated [13,14,108].

#### 3.1.4. Anti-IL-23 Antibodies in Dermatology

The effects of interleukin (IL)-12, IL-23 and IL-17 on tumorigenesis, particularly regarding their inhibition, has not been fully elucidated [118,119]. Generally, anti-IL-23 antibodies are associated with lower rates of serious adverse events compared with TNF-inhibitors, likely reflecting their more targeted immunologic mechanism [120]. They are used by dermatology to control psoriasis and are also utilised for psoriatic arthritis. The VOYAGE 1 and 2 trials did include patients with a history of malignancy, without recurrence, for at least 5 years. Within this group of 18 patients, only two malignancies were reported following guselkumab therapy [35].

Current evidence from large population-based studies and meta-analyses indicates that anti-IL-23 antibodies do not increase malignancy risk in patients with psoriasis or psoriatic arthritis, including those with prior cancer history [32,97,121,122]. Overall, the incidence of total malignancies, nonmelanoma skin cancers, and other site-specific cancers in patients receiving anti-IL-23 antibodies is comparable to rates seen with TNF-inhibitors and placebos [121]. Some analyses suggest a potentially lower risk of non-Hodgkin lymphoma, hepatobiliary cancer, and basal cell carcinoma with anti-IL-23 antibody therapy [32]. The joint guideline from the American Academy of Dermatology (AAD) and the National Psoriasis Foundation (NPF) states that, while long-term data on the effects of guselkumab and other anti-IL-23 antibodies on solid organ and lymphoreticular malignancies remain limited, no safety signals have been identified to date [97].

In summary, dermatology data do not demonstrate a signal for increased malignancy with IL-23 inhibition, and limited available data in patients with prior malignancy are reassuring though numbers remain small [32,35,121,122].

#### 3.1.5. Anti-IL-23 Antibodies in Gastroenterology

Anti-IL-23 antibodies are approved for moderate-to-severe CD and UC and are commonly used in patients refractory to TNF-inhibitor therapy [45,123,124,125].

Current evidence indicates that IL-23 inhibition does not increase incident malignancy risk in inflammatory bowel disease. Randomised controlled trials and meta-analyses report low rates of serious adverse events, including malignancy, with no signal of elevated cancer risk compared with placebo or other biologic classes [45,120,123,126].

Long-term data are emerging but are largely from open-label extensions as opposed to registry-level population-representative data for anti-IL-23 antibody agents in IBD. The LUCENT-3 open-label extension (to week 152) found that malignancy occurred in 0.3% of patients without concerning safety signals [40]. Risankizumab is being examined in the ongoing FORTIFY open-label extension (approximately 3 years reported) and 2 malignancies in total have been reported, excluding nonmelanomatous skin cancer [41]. The phase 3 GALAXI trials for guselkumab and CD have not indicated an increased risk for malignancy at induction or maintenance phases [127]. A further systematic review including patients with UC and CD demonstrated reduced serious adverse events and no increase in malignancy during both induction (12 week) or maintenance (52 week maximum) phases [128].

Anti-IL-23 inhibitors are relatively new agents in gastroenterology, and as such, robust registry data with detailed malignancy incidence are not yet available. Extrapolation from malignancy rates in the dermatology and rheumatology literature does not indicate an elevated risk [120]. The American Gastroenterological Association (AGA) notes that, while long-term population-level data remain limited, no malignancy-related safety concerns have been identified to date [129]. Importantly, although trial follow-up remains relatively short, there is no emerging signal of increased incident malignancy; however, data regarding cohorts including prior malignancy are lacking [40,45].

#### 3.1.6. Anti-IL-23 Antibodies in Rheumatology

Anti-IL-23 antibodies are incorporated into treatment algorithms for psoriatic arthritis but are generally not recommended for axial disease. Findings regarding malignancy across systematic reviews have been somewhat inconsistent. A 2019 systematic review and meta-analysis by Bilal et al. pooled results from 74 clinical trials and concluded that those exposed to biologic agents, compared to placebos, had an increased risk of opportunistic infections and malignancy [130]. They did not group the various biologic agents by class and included multiple rheumatologic conditions. They also included nonmelanomatous skin cancers with all other malignancies and grouped extension trials with randomised control trials. These factors may have inflated the malignancy risk for the rheumatology patients receiving these agents [130]. Collectively, the interpretation of malignancy risk is challenging as it may have been overstated [130].

Subsequently a meta-analysis of 48 randomised clinical trials, including studies in rheumatology, dermatology and gastroenterology populations, found no evidence of an association between anti-IL-23 antibodies and cancer incidence, with serious adverse events occurring infrequently. The review concluded that anti-IL-23 antibodies demonstrate a favourable overall safety profile with no signal for malignancy [131]. A comprehensive review, published in the Lancet, of anti-IL-23 antibodies across chronic inflammatory diseases, including psoriatic arthritis, found accumulating long-term safety data showing comparable rates of serious adverse events and infections to placebo, with no clear signal of increased malignancy risk [120].

When analysed by drug class rather than pooled across biologics, current evidence supports a favourable malignancy safety profile for IL-23 inhibitors in rheumatology [120,131].

#### 3.1.7. Anti-IL-17 Antibodies in Dermatology

IL-17 is a powerful pro-inflammatory cytokine and has been shown to contribute to the initiation, growth and proliferation of multiple malignancies [119]. Elevated levels of IL-17 have been associated with worse outcomes in several malignancies; therefore, extrapolating from this suggests that anti-IL-17 antibodies may reduce incident malignancy risk [119]. Kridin et al. reported that treatment with anti–IL-17 inhibitors was associated with a reduced incidence of several malignancies, including non-Hodgkin lymphoma, colorectal, hepatobiliary, and ovarian cancers, as well as melanoma and basal cell carcinoma [32]. It should be noted that this is incident malignancy in general populations, not recurrence in patients with prior cancer.

Registry data, including findings from Psonet, have not demonstrated an association between biologic therapy and incident malignancy, though the exact number of patients who received anti-IL-17 agents is not provided [19]. This study was limited by insufficient statistical power to assess and compare risks across specific agents or cancer types [19].

There are few studies which explore biologic prescribing in the setting of current or historical malignancy. One study by Battista et al. [132] retrospectively reviewed 20 adult patients, of whom 15 had a preceding diagnosis of malignancy prior to biologic treatment. The most frequently prescribed biologic agents were anti-IL-17 antibodies (47.7%) and anti-IL-23 antibodies (36.8%) which demonstrated sustained efficacy with a favourable safety profile [132]. Further evidence regarding incident malignancy includes a pooled analysis from 28 clinical trials of secukinumab and a post-marketing safety surveillance in psoriasis, psoriatic arthritis and ankylosing spondylitis patients which found that the rate of malignancy was consistent with previous reports, and more in-depth datasets for longer time periods did not increase malignancy numbers, indicating a favourable safety profile [36]. More recent reviews of the newer anti-IL-17 antibodies, bimekizumab, have shown no increased signal for malignancy as part of their 3-year extension trial [37].

Olivares-Guerrero et al. assessed the safety profile of biologic drugs used for psoriasis in those patients included in the Spanish Registry of Adverse Events of Biological Therapy (BIOBADADERM), compared to that of adalimumab [15]. The authors noted that ixekizumab, secukinumab and guselkumab were associated with a lower risk of benign, malignant and unspecified neoplasms compared to adalimumab [15]. A recent study with some data for Secukinumab found that incident malignancy rates were low and generally reassuring [133]. Papp et al. described the outcome from a multidisciplinary expert panel and concluded that those patients with psoriasis and a history of treated solid organ tumours with favourable prognosis have similar outcomes to those without solid organ tumours if treated with systemic biologic therapies. They highlighted that for individuals with a poor prognosis, the quality-of-life benefits of treating psoriasis may outweigh the theoretical risks [134]. Across dermatology data, IL-17 inhibition has not demonstrated an increased incident malignancy signal, and limited observational data in patients with prior solid organ malignancy are reassuring [132,134].

#### 3.1.8. Anti-IL-17 Antibodies in Rheumatology

Anti-IL-17 antibodies are generally used by rheumatologists to treat psoriatic arthritis and axial spondyloarthropathies. They were included in the systematic review by Bilal et al. which concluded that those exposed to biologic agents may be at increased risk of malignancy [130]. However, subgroup analyses for individual anti-IL-17 agents did not reach statistical significance, likely due to small sample sizes and short follow-ups [130]. Long-term extension trials and population-based studies in psoriasis and psoriatic arthritis, including cohorts with rheumatologic manifestations, show low exposure-adjusted malignancy rates with IL-17 inhibitors, with no signal of increasing risk with continued treatment [121]. The long-term safety of bimekizumab in adult patients with axial spondyloarthritis or psoriatic arthritis was evaluated in pooled results from integrated phase IIb/III clinical studies with a median exposure of 116 weeks. The authors reported most malignancies as singular events and no specific trends were observed, and demonstrated a reassuring safety profile, even at higher doses [38].

The brodalumab pharmacovigilance report, which included 7 years of data, found no new safety signals for malignancy with the crude rate of malignancy in 7 years of 1.01/100 patients, which was less than in PSOLAR (1.78/100 patients) [25,135]. Similarly, the data for ixekizumab are reassuring with observed malignancies in a pooled analysis of PSO and PSA data showing no increase incidence of cancer compared with the US population [39]. The 5-year post-marketing safety data for Secukinumab were also reassuring [136]. Longer-term, prospective pharmacovigilance data are lacking for the newer IL-17 inhibitors and safety is inferred from other agents and trial data.

Overall rheumatology data for IL-17 inhibitors do not indicate increased malignancy risk, and current guideline positions reflect this reassuring evidence base [108,121].

#### 3.1.9. Anti-IL-17 Antibodies in Gastroenterology

Anti-IL-17 antibodies are not used in gastroenterology as trials in CD showed clinical deterioration rather than improvement. Their blockade impairs mucosal barrier function and can enhance intestinal inflammation, making them unsuitable for IBD, and for this reason, anti-IL-17 antibodies are generally avoided or contraindicated [137].

#### 3.1.10. Ustekinumab in Dermatology

Ustekinumab targets the shared p40 subunit of both IL-12 and IL-23, both of which play an important role in carcinogenesis. IL-23, specifically, plays a vital role in gut tumorigenesis [138]. In the dermatology literature, PSOLAR data have shown no increased risk of malignancy with ustekinumab for individuals prescribed treatment for less than or greater than 12 months [95].

The AAD/NPF guidelines state that ustekinumab may be used in patients with a prior solid-tumour malignancy who have not responded to other therapies [97]. A 2012 study of cumulative safety data from four studies, with patients with psoriasis exposed to ustekinumab for up to 3 years, found that rates of malignancies, excluding nonmelanomatous skin cancer, were not increased and were in line with rates in the general US population [139]. A recent study found that ustekinumab showed a trend toward lower 5-year cancer risks compared with adalimumab, though this was not statistically significant when a 24-month lag period was included [133]. These data therefore support a reassuring malignancy safety profile for ustekinumab, including use in selected patients with prior solid organ malignancy [95,97].

#### 3.1.11. Ustekinumab in Rheumatology

Ustekinumab is used for psoriatic arthritis, systemic lupus erythematosus and giant cell arteritis [140]. There is a paucity of large, long-term ustekinumab studies focused exclusively on psoriatic arthritis, and registry data remain limited. A meta-analysis of 48 randomised trials in rheumatologic diseases reported that serious adverse events and malignancies were uncommon with anti–IL-23 therapies, including ustekinumab. While IL-12/23 p40 inhibitors showed slightly higher overall adverse event rates than IL-23 p19 agents, cancer incidence was not linked to IL-23 pathway blockade. Overall, ustekinumab and other IL-23 inhibitors demonstrated a favourable safety profile with no signal for increased malignancy risk [131]. The 3-year results from the PsABio real-world study of psoriatic arthritis noted 3/494 malignancies in the ustekinumab group and 4/557 in the anti-TNF group when a lag time of 1 year was applied, indicating a reassuring safety profile [21].

A study by Fiorentino et al. from the PSOLAR psoriasis registry found no increase in malignancy risk with ustekinumab in patients treated for over 12 months when compared to no ustekinumab treatment [95]. A 2019 study which integrated the 1-year safety data from 12 ustekinumab registrational trials included patients with moderate-severe psoriasis, psoriatic arthritis, or Crohn’s disease, and found low and comparable incidences of nonmelanomatous or solid organ malignancies with placebo groups after 1 year of treatment [141]. Overall, ustekinumab has a reassuring safety profile for rheumatologic conditions, though some data have been extrapolated from other inflammatory conditions.

Although rheumatology-specific registry data are smaller than for TNF-inhibitors, available evidence does not indicate increased malignancy risk with ustekinumab [21,95,131].

#### 3.1.12. Ustekinumab in Gastroenterology

Ustekinumab is used to treat IBD. The 5-year follow-up data in CD from the IM-UNITI ustekinumab trial noted that malignancy rates remained low, and concomitant therapies did not appear to affect ustekinumab’s safety or efficacy; however, the sample size may be insufficient to identify rare events [142]. A 2023 meta-analysis of head-head cohort trials found that ustekinumab had a reassuring safety profile, particularly compared to TNF-inhibitors [143]. IBD data were aggregated in a meta-analysis of 18 randomised controlled trials and explored anti-IL-23 antibodies, including ustekinumab, in moderate-severe CD [144]. It observed a lower risk of adverse events than placebos, but did not include malignancy or cancer incidence as a distinct endpoint or provide pooled data on malignancy risk [144].

The only data regarding the prior-malignancy cohort of patients, a retrospective cohort study of 390 patients with IBD and a history of cancer, found that exposure to ustekinumab or vedolizumab does not appear to be associated with an increased risk of subsequent new or recurrent cancer [145]. Regarding incident malignancy, a final pooled safety analysis of 2575 patients with IBD treated with ustekinumab found that malignancy events were not reported more frequently in ustekinumab treated patients [45]. Among IBD biologics, ustekinumab has some of the only datasets in patients with prior malignancy based on available retrospective cohort evidence [145].

#### 3.1.13. Tocilizumab in Rheumatology

Tocilizumab is used to treat RA, giant cell arteritis and juvenile idiopathic arthritis. An evaluation of the tocilizumab therapy in human cancers describes the potential use of this anti-IL-6 antibody to suppress IL-6 in tumour microenvironments, benefitting patients [146]. Current evidence indicates that tocilizumab is not associated with an increased malignancy risk in rheumatologic patients compared with DMARDs, TNF-inhibitors, or other biologic therapies. Large cohort studies and meta-analyses consistently show reassuring hazard ratios for invasive solid and hematologic cancers (excluding nonmelanomatous skin cancer) [23,147]. However, higher risk groups such as nursing home residents, patients with pre-existing malignancies, and rituximab users were excluded from that cohort study [147].

Integrated safety data from tocilizumab trials with a follow-up of mean 2.4 years did not show an increased risk of malignancy [46]. A further meta-analysis has found no apparent increase in the rate of malignancy compared to controls [148]. Data for 1496 RA patients followed for a mean 32 months from the French national registry “REGATE” found no additional risk factors for cancer [28]. Overall available data support a neutral malignancy risk profile for tocilizumab in rheumatology, though no dedicated prior-malignancy cohorts exist [23,46,147,148].

#### 3.1.14. Anti-Integrin Medications in Gastroenterology

Anti-integrin therapies such as vedolizumab and natalizumab block leukocyte trafficking into inflamed tissue, thereby reducing immune-driven inflammation. Current evidence indicates that anti-integrin therapies, including vedolizumab and natalizumab, are not associated with an increased malignancy risk in gastrointestinal diseases such as CD [149]. Meta-analyses and systematic reviews of randomised trials and observational cohorts consistently show no elevation in overall cancer, lymphoma, or skin cancer, compared with placebo or other biologics [149,150,151]. Pooled analyses likewise demonstrate no statistically significant increase in malignancy for either gut-selective agents (vedolizumab) or non-gut selective agents (natalizumab), with relative risks near 1 and broad confidence intervals [149,150].

Given their gut-selective mechanism, vedolizumab in particular, is frequently considered in patients where malignancy risk is a major concern, and available data support a reassuring safety profile [101,129,145].

#### 3.1.15. Anti-IL-4 and Anti-IL-13 Antibodies in Dermatology

Dupilumab and IL-13 pathway inhibitors have a generally reassuring malignancy safety profile in clinical trials, but observational studies raise specific concerns regarding cutaneous T-cell lymphoma (CTCL) and diagnostic unmasking of pre-existing disease rather than direct oncogenesis. Dupilumab is a human monoclonal IgG4 antibody that inhibits IL-4 and IL-13 signalling by binding to the IL-4Rα subunit, thereby blocking the inflammatory pathways central to type 2 immune-mediated diseases [152]. Dupilumab is used to treat moderate-to-severe atopic dermatitis in adults and children, as well as for prurigo nodularis and other type 2 inflammatory conditions [48,152].

Tralokinumab and lebrikizumab are both anti-IL-13 antibodies approved for moderate-to-severe atopic dermatitis in adults and adolescents [152]. The SOLO 1 and SOLO 2 studies for AD utilising dupilumab did not identify any signal for malignancy. The patient population had a median age of 36 and were followed up for 16 weeks [48]. A 5-year open-label extension study for patients diagnosed with AD did not find any association with malignancy [153]. Case series utilising dupilumab in patients with advanced solid organ cancer have indicated that AD can be safely controlled despite a malignancy diagnosis [154,155]. A pooled analysis of phase II and III placebo-controlled clinical trials of tralokinumab for moderate-to-severe AD did not show any increased risk of malignancy compared to placebo for up to 52 weeks [47]. A 2020 systematic review of anti-IL-13 antibodies highlighted an excellent safety profile [156].

Several population-based studies, pharmacovigilance reports, and systematic reviews have suggested a possible association between dupilumab treatment for atopic dermatitis and an elevated risk of cutaneous T-cell lymphoma (CTCL). CTCL can present in a clinically similar way to AD and is associated with the newer treatments for AD and anti-IL-13 antibodies are implicated in exacerbating or negatively impacting the severity of CTCL [157]. In a large retrospective cohort analysis, dupilumab exposure was linked to a markedly higher likelihood of developing CTCL, with most cases emerging more than a year after treatment initiation. No corresponding increase was observed for other types of cutaneous or lymphoid cancers [158]. These data support an association rather than proof of causation. Current evidence does not indicate that dupilumab or anti-IL-13 therapies exert a direct oncogenic effect, and the leading explanation is unmasking or acceleration of pre-existing malignant clones in patients misclassified as having AD or in whom early CTCL was not recognised [101,152]. Although data on anti-IL-13 agents such as tralokinumab remain limited, similar theoretical concerns exist due to their overlapping immunologic pathways.

A recent systematic review and expert consensus discussed clinical recommendations regarding CTCL and dupilumab therapy. When cutaneous T-cell lymphoma is confirmed by skin biopsy during dupilumab therapy, dupilumab should be discontinued immediately in all cases. If clinical features are non-aggressive, a 1–3 month “wait-and-see” approach with symptomatic treatment and close monitoring may be considered following discontinuation. If there is no improvement or is clinically worsening during this period, appropriate cutaneous T-cell lymphoma-directed treatment should then be initiated. In cases with features suggestive of aggressive disease, such treatment should be initiated without an observation period. If the diagnosis of CTCL is inconclusive, switching from dupilumab to methotrexate or phototherapy may be considered where appropriate. Switching to a Janus kinase inhibitor or to ciclosporin should be avoided [159].

Interestingly, the cytokine IL-13 has a positive impact on survival in colorectal cancer [160]. However, elevated IL-13Rα2 (a cell surface receptor that binds IL-13 with high affinity) expression is frequently linked to tumour invasiveness, advanced disease stage, and metastatic potential, contributing to a poorer prognosis in malignancies such as glioblastoma, colorectal cancer and breast cancer [18,54,98]. Further research is needed to tease out the link between IL-13, CTCL and other malignancies.

#### 3.1.16. Anti-IL-4/IL-13 Pathway Biologics in Respiratory Medicine

Anti-IL-4/Anti-IL-13 pathway biologics are utilised in respiratory medicine to treat severe asthma and, increasingly, COPD. In a large US cohort, dupilumab-treated patients demonstrated a higher incidence of lymphoma, particularly T/NK-cell lymphomas, compared with those on standard inhaled corticosteroid/long-acting β-agonist therapy. Despite this, overall cancer rates were unchanged, and all-cause mortality was lower among dupilumab users [30]. This is an incident malignancy signal, not a recurrence signal, and is possibly related to the same CTCL-unmasking mechanism discussed in the dermatology section. Randomised trials and meta-analyses in asthma and COPD populations have shown no increased risk of solid tumours or other hematologic cancers with dupilumab, and long-term data up to five years continue to support an overall favourable safety profile, aside from the observed lymphoma signal [30,161,162,163,164].

For tralokinumab and lebrikizumab, clinical trials and pooled analysis report no increased incidence of lymphoma or other malignancies, with no treatment-related cancers identified [165,166,167]. However, the trials included in the systematic reviews generally excluded patients with a prior malignancy, limiting conclusions in this subgroup [165]. Pre-clinical and clinical evidence reviewed by Braddock et al. found no evidence of elevated cancer risk associated with IL-13 pathway inhibition [168].

#### 3.1.17. Anti-IL-5 and IL-5R Therapies—Respiratory

Anti-IL-5 biologics (mepolizumab and reslizumab) and the IL-5 receptor antagonist benralizumab are widely used in severe eosinophilic asthma and have emerging roles in the management of COPD. Current evidence from randomised trials, meta-analyses, and post-marketing surveillance shows no increased malignancy risk associated with anti-IL-5 therapies for these conditions [50,169]. Large systematic reviews, including a Cochrane analysis, report no additional cancer risk compared with placebo, and long-term extension studies (up to 4.5 years) support a favourable and sustained safety profile [169,170]. A 5-year comparative safety analysis of benralizumab and mepolizumab noted small and similar numbers of malignancies across both studies [50]. These studies do not indicate increased malignancy risk with IL-5 pathway inhibition [50,169,170]. Although current evidence is reassuring, treatment decisions should be individualised in patients with a history of malignancy.

#### 3.1.18. Omalizumab in Respiratory Medicine

Omalizumab, an anti-IgE monoclonal antibody, is established in the management of severe allergic asthma and chronic spontaneous urticaria. Early pooled clinical trial data reported malignancies in 0.5% of omalizumab-treated patients compared with 0.2% of control subjects [51], leading to the inclusion of malignancy as a potential risk in the product label. However, subsequent pooled analysis in 2012 found no association between omalizumab treatment and malignancy risk, with a rate ratio below one implying that a causal link between omalizumab use and cancer development is unlikely [51]. Similarly, the EXCELS observational study, with a median follow-up of five years, found no significant difference in cancer incidence between omalizumab users and controls [22]. However, the follow-up duration was insufficient to account for cancers with a long latency period. Overall, omalizumab is considered safe with no malignancy signals in recent research [22,51].

#### 3.1.19. Omalizumab in Dermatology

Long-term data show no evidence of increased malignancy risk in patients with chronic urticaria treated with omalizumab. Large real-world and multicentre cohort studies with follow-up periods of up to eight years confirm its favourable long-term safety, with no treatment discontinuations or malignancy signals reported [33,171]. Furthermore, meta-analyses and systematic reviews of clinical trials and observational studies consistently indicate that omalizumab is well tolerated, with malignancy not identified as a safety concern [172,173].

Recent comprehensive reviews across all approved indications likewise conclude that cancer risk remains comparable to background rates, and post-marketing surveillance has revealed no new safety issues [174]. Overall contemporary evidence supports a neutral malignancy risk profile for omalizumab across respiratory and dermatologic indications [22,33,51,171,173,174].

### 3.2. Current Guidelines

#### 3.2.1. Dermatology

Regarding the dermatology guidelines, there are no cancer-specific recommendations provided or recommendations for particular biologics in the setting of malignancy. The AAD offers the most detailed discussion, stating that TNF-inhibitors are not associated with an increased malignancy risk and can be used in patients with a previous cancer diagnosis. The guideline supports ustekinumab in this setting, advises that further evidence is needed for IL-17 inhibitors, and does not recommend IL-23 inhibitors for patients with a history of malignancy [97].

In contrast, the EADV takes a more permissive view, noting that TNF-inhibitors, ustekinumab, and IL-17/23 inhibitors may be considered after consultation with oncology [96]. The BAD stresses individualised decision-making with multidisciplinary involvement but does not endorse or exclude specific biologic classes [175]. These differing positions underscore the complexity of managing psoriasis in patients with malignancy and highlight the need for clearer, more unified international guidance (Table 4 and Table 5).

#### 3.2.2. Gastroenterology

The ECCO, AGA, and ACG guidelines all provide structured, evidence-based assessments of incident malignancy risk associated with biologic therapy in inflammatory bowel disease [101,129,176]. ECCO offers the most detailed guidance, emphasising differential malignancy risks across therapeutic classes, highlighting the established association between thiopurines and lymphoma and noting the absence of a clear cancer signal with newer agents such as vedolizumab and ustekinumab [101]. The AGA similarly evaluates incident malignancy risk across biologics, concluding that anti-TNF agents carry a small but measurable lymphoma risk, whereas gut-selective and IL-12/23–targeted therapies have reassuring safety profiles [129]. The ACG guidelines reinforce these themes, underscoring the elevated incident cancer risk with thiopurines and the comparatively lower risk with targeted biologics, and advising individualised treatment in patients with current or prior malignancy [176]. Together, these guidelines consistently position vedolizumab and ustekinumab as preferred options when malignancy risk is a clinical concern (Table 4 and Table 5).

#### 3.2.3. Rheumatology

The EULAR, ACR, and BSR guidelines all address malignancy considerations in the use of biologic agents [108,177,178]. The 2024 EULAR “Points to Consider” indicate that current evidence regarding the use of TNF-inhibitors in patients with a prior history of malignancy is generally consistent and reassuring, demonstrating no significant increase in cancer recurrence compared with other therapeutic options. Nonetheless, EULAR advises that treatment decisions should be individualised, carefully considering the nature and timing of the previous cancer, the patient’s disease status, and made in collaboration with oncology specialists to ensure an informed and multidisciplinary approach to care [108]. They note particularly reassuring data for IL-17 and IL-12/23 inhibitors [108]. ACR similarly incorporates comparative malignancy evidence, noting no clear increased risk with newer non-TNF biologics [177]. BSR emphasises the favourable malignancy profile of IL-17 and IL-12/23 inhibitors [178]. Collectively, these guidelines support targeted non-TNF biologics when malignancy risk is a key clinical concern. Rituximab can also be used for RA when lymphoma risk is a concern (Table 4 and Table 5).

#### 3.2.4. Respiratory Medicine

The 2026 Global Initiative for Asthma (GINA) [179,180], American Thoracic Society/European Respiratory Society (ATS/ERS) [181], and British Thoracic Society (BTS) [182] severe asthma guidelines provide detailed recommendations on the use of biologic therapies but do not explicitly address malignancy risk or provide guidance for patients with a cancer history. All three documents focus on efficacy, eligibility criteria, and general safety monitoring, without commenting on long-term oncologic outcomes. As a result, clinicians must rely on trial data and post-marketing evidence, rather than formal respiratory guidelines, when considering biologic therapy in patients with prior malignancy (Table 4 and Table 5).

## 4. Discussion

The studies reported in this review highlight the challenges for clinicians caring for patients with two of the most common non-communicable illnesses in society. Current evidence is generally insufficient to definitively distinguish between biologic drug classes, as apparent differences often reflect significant variations in follow-up duration and data availability. While newer classes like IL-17 and IL-23 inhibitors show reassuring safety profiles, they lack the extensive, long-term registry data available for TNF-inhibitors, which have been used for decades. Despite a wealth of studies and peer-reviewed publications, many wrestle with concerns that effectively managing one condition will compromise outcomes for the other. Their concerns are compounded by lack of long follow-up periods and heterogeneous patient populations in the available data in this area [52]. These concerns are tempered by the knowledge that modern biologic treatments have radically improved outcomes for patients, and by their lived professional experience witnessing this transformation in quality of survival. Guidelines for dermatology [96,97,175], rheumatology [177,178] and gastroenterology [101,176,183] recommend discussion with oncology colleagues in the setting of a newly diagnosed or historic malignancy. Inter-specialty collaboration, attention to emerging data, and participation in large-scale pharmacovigilance registries are essential to further our understanding of this complex area and to better treat our patients.

Some patients may have been treated with historical therapies, like thiopurines, which have contributed to perceived elevated malignancy risk [184]. In addition, some chronic inflammatory conditions may be associated with an increased risk of malignancy. For instance, a meta-analysis of 112 studies involving more than two million individuals with psoriasis or psoriatic arthritis reported a modest increase in overall incident cancer risk among patients with psoriasis, with a relative risk of 1.21 [94]. However, appropriate and effective disease control for inflammatory conditions improves the symptoms of the affected tissue, positively affects systemic inflammation, and contributes to overall wellbeing [19,34,140,185,186].

The challenges in providing reassurance to patients and prescribers outlined in this review are evolving. The proportion of younger patients (less than 50 years old) diagnosed with malignancy is increasing globally; as a result, the number of patients prescribed these agents in the setting of cancer will undoubtedly increase, and the duration of exposure to therapy will be longer [187], reinforcing the challenges raised in this review. In parallel, the global population is ageing, and as illustrated in Figure 1, the variety of biologic therapies available is increasing, as are the number of patients with cancer-related comorbidities and those living beyond cancer treatment [188]. Equally it must be emphasised that the impact on a patient’s quality of life from uncontrolled psoriasis, RA or IBD is significant [189], highlighting the need for improved guidance in this oncology: an autoimmune inflammatory disease interface.

This review has highlighted that reassuring data are emerging from pharmacovigilance registries and large prospective studies including many inflammatory conditions [25,95]. Studies in this area are also evolving, with, firstly, the inclusion of patient partners in such studies—a development that has already transformed cancer research [190,191]—and secondly, with the advent of artificial intelligence-enabled data analysis with the potential for digital twin development to help guide personalised treatment strategies [192,193,194]. However, a key challenge in enabling such advances is data integration and the need for ancestral, oncologic and inflammatory disease-related diversity in these datasets [195]. To address this, we propose prioritising that longitudinal registry and real-world databases become a condition of regulatory drug approval. Such prospectively maintained digitally enabled databases would help address many of the concerns highlighted for clinicians and patients in the paper, concerns that will increase as the range and diversity of biologic therapies expand for inflammatory diseases. Precedence for such registries exist such as that maintained in Ireland during the COVID-19 pandemic [196] and has been proposed by other groups for cancer therapeutics [197].

## 5. Conclusions

This review has described the safety profiles of many biologic agents regarding malignancy risk and has examined data across specialties. In general, the available observational data suggest that biologic agents carry a lower malignancy risk than older immunosuppressants or conventional disease-modifying agents, although direct comparative trials are lacking. Several biologic classes, including TNF-inhibitors, anti-IL-17, and anti-IL-23 agents, appear to be associated with a modest increase in nonmelanoma skin cancer risk in observational cohorts. The evidence base throughout this review pertains predominantly to patients with prior, treated, and currently inactive malignancy, and that extrapolation to patients with active or recently treated malignancy should be made with caution given the near-total exclusion of such patients from the studies cited.

When a patient with malignancy and a chronic immune-mediated inflammatory disease requires systemic therapy, several considerations apply. TNF-inhibitors may warrant caution in the context of an active immune-sensitive cancer such as lymphoma, but data from rheumatology cohorts and the EULAR do not demonstrate a significant increase in recurrence risk for solid organ cancers. This provides some reassurance, albeit from observational studies with limited power to detect small effects. Among individual agents, newer biologics such as anti-IL-17 and anti-IL-23 agents have less long-term follow-up data, though preclinical and post-marketing pharmacovigilance data have not raised safety concerns to date. Anti-IL-23 agents have not been associated with malignancy recurrence in the small number of patients with a prior history of malignancy studied so far, though these findings are based on limited sample sizes and should be interpreted with caution rather than taken as evidence of a protective or risk-reducing effect. There is a concern regarding dupilumab and the potential diagnostic ‘unmasking’ of pre-existing CTCL or association with CTCL, and further exploration is warranted. There is a clear need for validated decision frameworks that incorporate cancer type, stage, prior therapies, and latency, alongside the clinical impact of chronic immune-mediated diseases.

We suggest mandatory engagement with real-world registries and databases as a condition of approval for new and established biologic agents, supported by a pragmatic monitoring approach. Such post-marketing surveillance provides the most clinically relevant information regarding true malignancy risks and will assist practicing clinicians to address the concerns of their patients and their colleagues. Appropriately treating the complex clinical situation of patients with inflammatory and immune-mediated disease with a concomitant diagnosis of malignancy is an essential focus of future research and clinical guidelines.

## Figures and Tables

**Table 1:** Biologic agents (Derm, Rheum, Resp, Gastro) approved in Ireland.

5-Year Period	Drug	Approval Year	Specialty Area(s)
1995–1999	Rituximab	1998	Rheum/Derm
1995–1999	Infliximab	1999	Rheum/Gastro/Derm
2000–2004	Etanercept	2000	Rheum/Derm
2000–2004	Anakinra	2002	Rheum
2000–2004	Adalimumab	2003	Rheum/Gastro/Derm
2005–2009	Omalizumab	2005	Resp/Derm
2005–2009	Abatacept	2007	Rheum
2005–2009	Ustekinumab	2009	Derm/Rheum/Gastro
2005–2009	Tocilizumab	2009	Rheum
2005–2009	Certolizumab pegol	2009	Rheum/Derm
2005–2009	Golimumab	2009	Rheum/Gastro
2010–2014	Belimumab	2011	Rheum
2010–2014	Vedolizumab	2014	Gastro
2015–2019	Secukinumab	2015	Derm/Rheum
2015–2019	Mepolizumab	2015	Resp/Rheum
2015–2019	Ixekizumab	2016	Derm/Rheum
2015–2019	Reslizumab	2016	Resp
2015–2019	Brodalumab	2017	Derm
2015–2019	Guselkumab	2017	Derm/Rheum/Gastro
2015–2019	Dupilumab	2017	Derm/Resp
2015–2019	Sarilumab	2017	Rheum
2015–2019	Benralizumab	2018	Resp
2015–2019	Tildrakizumab	2018	Derm
2015–2019	Risankizumab	2019	Derm/Rheum/Gastro
2020–2024	Bimekizumab	2021	Derm/Rheum
2020–2024	Tralokinumab	2021	Derm
2020–2024	Anifrolumab	2022	Rheum
2020–2024	Spesolimab	2022	Derm
2020–2024	Tezepelumab	2022	Resp
2020–2024	Mirikizumab	2023	Gastro
2020–2024	Lebrikizumab	2023	Derm
2025–2029	Nemolizumab	2025	Derm

**Table 4:** Summary of Clinical Guidelines on Biologics and Malignancy Risk.

Organisation/Guideline	Year	Target Disease Population	Formal Evidence Grading Used	Level of Evidence for Malignancy-Related Recommendation	Key Recommendations and Positions on Malignancy
AAD (American Academy of Dermatology)/National Psoriasis Foundation	2019	Adults with moderate-to-severe plaque psoriasis	Yes—evidence-based model with lettered grades (A–C)	Grade B/C for malignancy-related statements, no dedicated systematic review of cancer outcomes	States TNF-inhibitors are not associated with increased malignancy risk and can be used in patients with a previous cancer diagnosis. Supports ustekinumab in this setting, advises that further evidence is needed for interleukin-17 inhibitors, and does not recommend interleukin-23 inhibitors for patients with a history of malignancy.
EADV/EuroGuiDerm (European Academy of Dermatology and Venereology, in cooperation with the European Dermatology Forum)	2020–2021	Adults with moderate-to-severe psoriasis vulgaris, including those with a history of malignancy	Yes—S3-level, evidence- and consensus-based guideline with formal levels of evidence and consensus voting	Conditional/‘suggest’-strength recommendations, explicitly noting a lack of long-term experience for newer agents	Takes a more permissive view, suggesting TNF-inhibitors and ustekinumab can be used based on existing safety data, and that interleukin-17 and interleukin-23 inhibitors can be considered on a case-by-case basis despite limited long-term experience, generally in consultation with oncology.
BAD (British Association of Dermatologists)	2017 (2020 rapid update)	Adults with moderate-to-severe plaque psoriasis	Yes—graded recommendations with strength of evidence	Not specifically graded for the malignancy/biologic-class question, framed as general principle rather than a graded recommendation	Emphasises individualised decision-making and multidisciplinary involvement when treating patients with a history of malignancy but does not explicitly endorse or exclude specific biologic classes.
ECCO (European Crohn’s and Colitis Organisation)	2023	Adults with inflammatory bowel disease (Crohn’s disease and ulcerative colitis), including those with current or prior malignancy	Yes—formal evidence levels (EL1–EL5) with consensus percentages for each statement	Evidence level 2–3 for thiopurine-lymphoma association, lower evidence levels (EL4–EL5) for reassurance on vedolizumab and ustekinumab, reflecting an absence of signal rather than confirmed safety	Provides the most detailed guidance among the gastroenterology guidelines, highlighting the established association between thiopurines and lymphoma while noting the absence of a clear cancer signal with newer agents such as vedolizumab and ustekinumab.
AGA (American Gastroenterological Association) Clinical Practice Update	2024	Adults with inflammatory bowel disease and current or prior malignancy	No—a Clinical Practice Update/Commentary, which AGA designates as expert-opinion guidance rather than a formally GRADE-graded clinical practice guideline	Not graded, statements are framed as expert ‘Best Practice Advice’	Evaluates malignancy risk across biologic classes, concluding that anti-TNF-inhibitor agents carry a small but measurable lymphoma risk, whereas vedolizumab and interleukin-12/23-targeted therapies such as ustekinumab have reassuring safety profiles, though long-term data remain limited.
ACG (American College of Gastroenterology), Preventive Care in Inflammatory Bowel Disease	2025 (updates 2017)	Adults with inflammatory bowel disease, including health-maintenance and malignancy-screening considerations	Yes—GRADE-based recommendations, with expert-consensus ‘key concept statements’ used where formal grading was not possible	Mix of graded recommendations and ungraded key concept statements depending on the topic	Underscores the elevated cancer risk associated with thiopurines compared with the comparatively lower risk seen with targeted biologics and advises individualised treatment and cancer screening in patients with current or prior malignancy.
EULAR (European Alliance of Associations for Rheumatology), Points to Consider on the Initiation of Targeted Therapies in Patients with a History of Cancer	2024 (publ. 2025)	Adults with inflammatory arthritis (rheumatoid arthritis, psoriatic arthritis, and spondyloarthritis) and a history of malignancy	Yes—formal EULAR ‘points to consider’ process, underpinned by a systematic literature review and meta-analysis, with levels of evidence and levels of agreement	High levels of evidence and agreement reported for most points, based on a meta-analysis of 15 studies (4428 patients/15,062 patient-years exposed vs. 13,698 patients/41,160 patient-years unexposed, overall hazard ratio 0.90, 95% confidence interval 0.74–1.10)	States that current evidence on TNF-inhibitor in patients with a prior history of malignancy is generally consistent and reassuring, showing no significant increase in cancer recurrence compared with other therapeutic options, while advising individualised, oncology-collaborative decision-making. Notes particularly reassuring data for interleukin-17 and interleukin-12/23 inhibitors.
ACR (American College of Rheumatology), Guideline for the Treatment of Rheumatoid Arthritis	2021	Adults with rheumatoid arthritis, including those with comorbidities and high-risk populations	Yes—GRADE methodology, with recommendations graded as strong or conditional	Predominantly conditional recommendations (77% of all recommendations), reflecting moderate-to-low certainty evidence for most comparative safety questions	Incorporates comparative evidence on biologic and targeted synthetic disease-modifying drugs, showing no clear increased malignancy risk with newer non-TNF-inhibitor biologic therapies compared with TNF-inhibitor.
BSR (British Society for Rheumatology), Biologic DMARD Safety Guideline in Inflammatory Arthritis	2019 (update pending)	Adults with inflammatory arthritis (rheumatoid arthritis, psoriatic arthritis, and axial spondyloarthritis)	Yes -GRADE-based grading of recommendations with strength of agreement	Graded recommendations (for example, grade 2C-type notation) reflecting generally low-to-moderate certainty evidence for malignancy-specific statements	Emphasises the lymphoma risk associated with TNF-inhibitors and highlights the comparatively favourable malignancy profile of interleukin-17 and interleukin-12/23 inhibitors.
GINA (Global Initiative for Asthma), Global Strategy for Asthma Management and Prevention	2026	Adults, adolescents, and children with asthma, including severe/biologic-eligible phenotypes	No -GINA does not conduct its own GRADE-based reviews, recommendations are based on expert review of the literature, including externally conducted GRADE-based systematic reviews where available	Not formally graded as a whole document, individual cited studies vary in quality	Provides detailed recommendations on the use of biologic therapies for severe asthma but does not explicitly address malignancy risk or provide guidance for patients with a cancer history.
ATS/ERS (American Thoracic Society/European Respiratory Society), Severe Asthma Guideline	2020	Adults and children with severe asthma being considered for biologic therapy	Yes—GRADE approach used to formulate questions, grade evidence, and develop recommendations	Conditional recommendations, generally based on low-to-moderate certainty evidence	Provides GRADE-based recommendations on the use of anti-interleukin-5, anti-immunoglobulin E, and related therapies for severe asthma, focused on efficacy and biomarker-guided selection, without addressing malignancy risk or cancer history.
BTS/NICE/SIGN, Asthma: Diagnosis, Monitoring and Chronic Asthma Management	2024 (amends 2019)	Adults, young people, and children with asthma, including difficult and severe asthma	Yes—NICE/SIGN evidence-grading methodology applied to systematic evidence reviews	Recommendations vary in strength depending on underlying evidence quality, sections on difficult and severe asthma noted as under review at the time of the 2024 amendment	Provides recommendations on specialist therapies, including biologics, for severe asthma, focusing on eligibility, efficacy, and general safety monitoring, without explicit guidance on malignancy risk or biologic selection in patients with a cancer history.
AAD (American Academy of Dermatology)/National Psoriasis Foundation	2019	Adults with moderate-to-severe plaque psoriasis	Yes—evidence-based model with lettered grades (A–C)	Grade B/C for malignancy-related statements, no dedicated systematic review of cancer outcomes	States TNF-inhibitors are not associated with increased malignancy risk and can be used in patients with a previous cancer diagnosis. Supports ustekinumab in this setting, advises that further evidence is needed for interleukin-17 inhibitors, and does not recommend interleukin-23 inhibitors for patients with a history of malignancy.
EADV/EuroGuiDerm (European Academy of Dermatology and Venereology, in cooperation with the European Dermatology Forum)	2020–2021	Adults with moderate-to-severe psoriasis vulgaris, including those with a history of malignancy	Yes—S3-level, evidence- and consensus-based guideline with formal levels of evidence and consensus voting	Conditional/‘suggest’-strength recommendations, explicitly noting a lack of long-term experience for newer agents	Takes a more permissive view, suggesting TNF-inhibitors and ustekinumab can be used based on existing safety data, and that interleukin-17 and interleukin-23 inhibitors can be considered on a case-by-case basis despite limited long-term experience, generally in consultation with oncology.
BAD (British Association of Dermatologists)	2017 (2020 rapid update)	Adults with moderate-to-severe plaque psoriasis	Yes—graded recommendations with strength of evidence	Not specifically graded for the malignancy/biologic-class question, framed as general principle rather than a graded recommendation	Emphasises individualised decision-making and multidisciplinary involvement when treating patients with a history of malignancy but does not explicitly endorse or exclude specific biologic classes.
ECCO (European Crohn’s and Colitis Organisation)	2023	Adults with inflammatory bowel disease (Crohn’s disease and ulcerative colitis), including those with current or prior malignancy	Yes—formal evidence levels (EL1–EL5) with consensus percentages for each statement	Evidence level 2–3 for thiopurine-lymphoma association, lower evidence levels (EL4–EL5) for reassurance on vedolizumab and ustekinumab, reflecting an absence of signal rather than confirmed safety	Provides the most detailed guidance among the gastroenterology guidelines, highlighting the established association between thiopurines and lymphoma while noting the absence of a clear cancer signal with newer agents such as vedolizumab and ustekinumab.
AGA (American Gastroenterological Association) Clinical Practice Update	2024	Adults with inflammatory bowel disease and current or prior malignancy	No—a Clinical Practice Update/Commentary, which AGA designates as expert-opinion guidance rather than a formally GRADE-graded clinical practice guideline	Not graded, statements are framed as expert ‘Best Practice Advice’	Evaluates malignancy risk across biologic classes, concluding that anti-TNF-inhibitor agents carry a small but measurable lymphoma risk, whereas vedolizumab and interleukin-12/23-targeted therapies such as ustekinumab have reassuring safety profiles, though long-term data remain limited.
ACG (American College of Gastroenterology), Preventive Care in Inflammatory Bowel Disease	2025 (updates 2017)	Adults with inflammatory bowel disease, including health-maintenance and malignancy-screening considerations	Yes—GRADE-based recommendations, with expert-consensus ‘key concept statements’ used where formal grading was not possible	Mix of graded recommendations and ungraded key concept statements depending on the topic	Underscores the elevated cancer risk associated with thiopurines compared with the comparatively lower risk seen with targeted biologics and advises individualised treatment and cancer screening in patients with current or prior malignancy.
EULAR (European Alliance of Associations for Rheumatology), Points to Consider on the Initiation of Targeted Therapies in Patients with a History of Cancer	2024 (publ. 2025)	Adults with inflammatory arthritis (rheumatoid arthritis, psoriatic arthritis, and spondyloarthritis) and a history of malignancy	Yes—formal EULAR ‘points to consider’ process, underpinned by a systematic literature review and meta-analysis, with levels of evidence and levels of agreement	High levels of evidence and agreement reported for most points, based on a meta-analysis of 15 studies (4428 patients/15,062 patient-years exposed vs. 13,698 patients/41,160 patient-years unexposed, overall hazard ratio 0.90, 95% confidence interval 0.74–1.10)	States that current evidence on TNF-inhibitor in patients with a prior history of malignancy is generally consistent and reassuring, showing no significant increase in cancer recurrence compared with other therapeutic options, while advising individualised, oncology-collaborative decision-making. Notes particularly reassuring data for interleukin-17 and interleukin-12/23 inhibitors.
ACR (American College of Rheumatology), Guideline for the Treatment of Rheumatoid Arthritis	2021	Adults with rheumatoid arthritis, including those with comorbidities and high-risk populations	Yes—GRADE methodology, with recommendations graded as strong or conditional	Predominantly conditional recommendations (77% of all recommendations), reflecting moderate-to-low certainty evidence for most comparative safety questions	Incorporates comparative evidence on biologic and targeted synthetic disease-modifying drugs, showing no clear increased malignancy risk with newer non-TNF-inhibitor biologic therapies compared with TNF-inhibitor.
BSR (British Society for Rheumatology), Biologic DMARD Safety Guideline in Inflammatory Arthritis	2019 (update pending)	Adults with inflammatory arthritis (rheumatoid arthritis, psoriatic arthritis, and axial spondyloarthritis)	Yes -GRADE-based grading of recommendations with strength of agreement	Graded recommendations (for example, grade 2C-type notation) reflecting generally low-to-moderate certainty evidence for malignancy-specific statements	Emphasises the lymphoma risk associated with TNF-inhibitors and highlights the comparatively favourable malignancy profile of interleukin-17 and interleukin-12/23 inhibitors.
GINA (Global Initiative for Asthma), Global Strategy for Asthma Management and Prevention	2026	Adults, adolescents, and children with asthma, including severe/biologic-eligible phenotypes	No -GINA does not conduct its own GRADE-based reviews, recommendations are based on expert review of the literature, including externally conducted GRADE-based systematic reviews where available	Not formally graded as a whole document, individual cited studies vary in quality	Provides detailed recommendations on the use of biologic therapies for severe asthma but does not explicitly address malignancy risk or provide guidance for patients with a cancer history.
ATS/ERS (American Thoracic Society/European Respiratory Society), Severe Asthma Guideline	2020	Adults and children with severe asthma being considered for biologic therapy	Yes—GRADE approach used to formulate questions, grade evidence, and develop recommendations	Conditional recommendations, generally based on low-to-moderate certainty evidence	Provides GRADE-based recommendations on the use of anti-interleukin-5, anti-immunoglobulin E, and related therapies for severe asthma, focused on efficacy and biomarker-guided selection, without addressing malignancy risk or cancer history.
BTS/NICE/SIGN, Asthma: Diagnosis, Monitoring and Chronic Asthma Management	2024 (amends 2019)	Adults, young people, and children with asthma, including difficult and severe asthma	Yes—NICE/SIGN evidence-grading methodology applied to systematic evidence reviews	Recommendations vary in strength depending on underlying evidence quality, sections on difficult and severe asthma noted as under review at the time of the 2024 amendment	Provides recommendations on specialist therapies, including biologics, for severe asthma, focusing on eligibility, efficacy, and general safety monitoring, without explicit guidance on malignancy risk or biologic selection in patients with a cancer history.

**Table 5:** Summary table describing the relevant observations for each biologic agent.

Biologic Agent/ Drug Class	Primary Condition(s) Treated	Observed Malignancy Signals	Evidence from Prior Malignancy Cohorts	Guideline Recommendations Regarding Malignancy
TNF-Inhibitors	Psoriasis, Inflammatory Bowel Disease (Crohn’s disease, Ulcerative colitis), Rheumatoid Arthritis (RA), Psoriatic Arthritis (PsA), Spondyloarthropathies	Nonmelanomatous skin cancer (NMSC), melanoma, and lymphomas. Potential lung cancer signal with >12 months exposure. Lymphoma risk in IBD often associated with concomitant thiopurines. No significant increase in overall malignancy across major registers (RABBIT, BSRBR, ARTIS, DANBIO).	“No increased incident malignancy in general biologic-naive registry populations” and “No increased recurrence risk in patients with prior malignancy (BSRBR, EULAR meta-analysis).” No prospective or randomised studies available.	AAD: Can be used in patients with prior cancer. EADV: May be considered after oncology consultation. AGA: Small but measurable lymphoma risk. ECCO: Absence of clear signal with monotherapy. EULAR (2024): Recommended for patients with history of solid organ malignancy.
Ustekinumab (IL-12/23)	Psoriasis, PsA, Crohn’s Disease, UC	No increased risk in PSOLAR or IM-UNITI; trend toward lower 5-year risk compared to adalimumab.	Retrospective IBD cohorts show no association with subsequent new or recurrent cancer.	AAD: May be used in patients with prior solid-tumor malignancy. AGA/ECCO: Preferred option for high-risk patients.
Anti-IL-23 Antibodies	Psoriasis, Psoriatic Arthritis, Crohn’s disease, Ulcerative colitis	No safety signals identified; rates comparable to placebo and TNF-inhibitors in clinical trials (LUCENT-3, FORTIFY, GALAXI).	VOYAGE 1/2 trials included 18 patients with prior malignancy (2 cases reported post-treatment). Current data do not suggest excess recurrence risk in limited cohorts.	AAD/NPF: Does not currently recommend for patients with prior malignancy due to limited long-term data. AGA/ECCO: Positioned as preferred options when malignancy risk is a concern.
Anti-IL-17 Antibodies	Psoriasis, PsA, Axial Spondyloarthritis	Neutral or potentially reduced risk of certain cancers (NMSC, melanoma) in some studies; generally reassuring safety profile.	Limited observational data in patients with prior solid organ malignancy are reassuring.	AAD: Further evidence needed. EULAR: Notes particularly reassuring data for IL-17 inhibitors.
Anti-Integrin (Vedolizumab)	Inflammatory Bowel Disease	No elevation in overall cancer, lymphoma, or skin cancer due to gut-selective mechanism.	Retrospective studies (390 patients) show no increased risk of new or recurrent cancer.	AGA/ECCO: Frequently considered preferred in patients where malignancy risk is a major concern.
Anti-IL-4/Anti-IL-13 (Dupilumab)	Atopic Dermatitis, Asthma, COPD	Potential association with cutaneous T-cell lymphoma (CTCL) unmasking; asthma cohort showed increased T/NK-cell lymphoma.	Case series in advanced solid organ cancer show atopic dermatitis can be safely controlled.	GINA/ATS/ERS: Do not explicitly address malignancy risk; focus on efficacy.
Omalizumab (Anti-IgE)	Asthma, Chronic Spontaneous Urticaria	Historical 0.5% vs. 0.2% trial signal led to label warning; subsequent pooled analyses (EXCELS) found no causal link.	-	Neutral risk profile supported by contemporary evidence.
Anti-IL-5 Antibodies	Asthma, COPD	No increased risk observed in randomized trials or meta-analyses.	-	Not explicitly addressed in severe asthma guidelines.
Tocilizumab (anti-IL-6 antibody)	RA, giant cell arteritis and juvenile idiopathic arthritis	Large cohort studies and meta-analyses found reassuring safety data.	-	EULAR: No indication that there is an increased risk of malignancy compared to anti-TNF agents.

## Data Availability

All data used in this review are publicly available.

## Abbreviations

The following abbreviations are used in this manuscript:

AAD	American Academy of Dermatology
ACG	American College of Gastroenterology
ACR	American College of Rheumatology
AD	Atopic Dermatitis
AGA	American Gastroenterological Association
ANCA	Anti-neutrophil cytoplasmic antibodies
ARTIS	Anti-Rheumatic Therapy in Sweden
ATS/ERS	American Thoracic Society/European Respiratory Society
BAD	British Association of Dermatologists
bDMARD	Biologic Disease-Modifying Antirheumatic Drug
BSR	British Society for Rheumatology
BSRBR	British Society for Rheumatology Biologics Register
BTS	British Thoracic Society
CD	Crohn’s Disease
COPD	Chronic Obstructive Pulmonary Disease
CTCL	Cutaneous T-cell Lymphoma
DMARD	Disease-Modifying Antirheumatic Drug
EADV	European Academy of Dermatology and Venereology
ECCO	European Crohn’s and Colitis Organisation
EULAR	European Alliance of Associations for Rheumatology
GINA	Global Initiative for Asthma
IBD	Inflammatory Bowel Disease
IgE	Immunoglobulin E
IL	Interleukin
NPF	National Psoriasis Foundation
NXP2	Nuclear matrix protein 2 (an autoantibody)
PsA	Psoriatic Arthritis
PSOLAR	Psoriasis Longitudinal Assessment and Registry
PUVA	Psoralen plus Ultraviolet A
RA	Rheumatoid Arthritis
SLE	Systemic Lupus Erythematosus
TIF1-γ	Transcription intermediary factor 1-gamma (an autoantibody)
TNF	Tumour Necrosis Factor
UC	Ulcerative Colitis

## References

[1] Trafford A.M., Parisi R., Kontopantelis E., Griffiths C.E.M., Ashcroft D.M. (2019). Association of Psoriasis with the Risk of Developing or Dying of Cancer: A Systematic Review and Meta-analysis. JAMA Dermatol..

[2] Coussens L.M., Werb Z. (2002). Inflammation and cancer. Nature.

[3] Boffetta P., Gridley G., Lindelöf B. (2001). Cancer risk in a population-based cohort of patients hospitalized for psoriasis in Sweden. J. Investig. Dermatol..

[4] Brauchli Y.B., Jick S.S., Miret M., Meier C.R. (2009). Psoriasis and risk of incident cancer: An inception cohort study with a nested case-control analysis. J. Investig. Dermatol..

[5] Chen Y.J., Wu C.Y., Chen T.J., Shen J.L., Chu S.Y., Wang C.B., Chang Y.T. (2011). The risk of cancer in patients with psoriasis: A population-based cohort study in Taiwan. J. Am. Acad. Dermatol..

[6] Chiesa Fuxench Z.C., Shin D.B., Ogdie Beatty A., Gelfand J.M. (2016). The Risk of Cancer in Patients with Psoriasis: A Population-Based Cohort Study in the Health Improvement Network. JAMA Dermatol..

[7] Frentz G., Olsen J.H. (1999). Malignant tumours and psoriasis: A follow-up study. Br. J. Dermatol..

[8] Gelfand J.M., Berlin J., Van Voorhees A., Margolis D.J. (2003). Lymphoma rates are low but increased in patients with psoriasis: Results from a population-based cohort study in the United Kingdom. Arch. Dermatol..

[9] Gelfand J.M., Shin D.B., Neimann A.L., Wang X., Margolis D.J., Troxel A.B. (2006). The risk of lymphoma in patients with psoriasis. J. Investig. Dermatol..

[10] Margolis D., Bilker W., Hennessy S., Vittorio C., Santanna J., Strom B.L. (2001). The risk of malignancy associated with psoriasis. Arch. Dermatol..

[11] Paul C.F., Ho V.C., McGeown C., Christophers E., Schmidtmann B., Guillaume J.C., Lamarque V., Dubertret L. (2003). Risk of malignancies in psoriasis patients treated with cyclosporine: A 5 y cohort study. J. Investig. Dermatol..

[12] Papp K.A., Strober B., Augustin M., Calabro S., Londhe A., Chevrier M. (2012). PSOLAR: Design, utility, and preliminary results of a prospective, international, disease-based registry of patients with psoriasis who are receiving, or are candidates for, conventional systemic treatments or biologic agents. J. Drugs Dermatol..

[13] Dixon W.G., Watson K.D., Lunt M., Mercer L.K., Hyrich K.L., Symmons D.P. (2010). Influence of anti-tumor necrosis factor therapy on cancer incidence in patients with rheumatoid arthritis who have had a prior malignancy: Results from the British Society for Rheumatology Biologics Register. Arthritis Care Res..

[14] Strangfeld A., Hierse F., Rau R., Burmester G.R., Krummel-Lorenz B., Demary W., Listing J., Zink A. (2010). Risk of incident or recurrent malignancies among patients with rheumatoid arthritis exposed to biologic therapy in the German biologics register RABBIT. Arthritis Res. Ther..

[15] Olivares-Guerrero M., Daudén E., Riera-Monroig J., González-Quesada A., Sahuquillo-Torralba A., Rivera-Díaz R., Carrascosa J.M., Belinchón I., Gómez-García F.J., Herrera-Acosta E. (2025). Risk of adverse events of psoriasis treatment with biologic agents and new small molecules—BIOBADADERM Registry. J. Eur. Acad. Dermatol. Venereol..

[16] Hellgren K., Dreyer L., Arkema E.V., Glintborg B., Jacobsson L.T., Kristensen L.E., Feltelius N., Hetland M.L., Askling J., ARTIS Study Group (2017). Cancer risk in patients with spondyloarthritis treated with TNF inhibitors: A collaborative study from the ARTIS and DANBIO registers. Ann. Rheum. Dis..

[17] Lichtenstein G.R., Feagan B.G., Cohen R.D., Salzberg B.A., Diamond R.H., Langholff W., Londhe A., Sandborn W.J. (2014). Drug therapies and the risk of malignancy in Crohn’s disease: Results from the TREAT™ Registry. Am. J. Gastroenterol..

[18] Menter A., Thaçi D., Wu J.J., Abramovits W., Kerdel F., Arikan D., Guo D., Ganguli A., Bereswill M., Camez A. (2017). Long-Term Safety and Effectiveness of Adalimumab for Moderate to Severe Psoriasis: Results from 7-Year Interim Analysis of the ESPRIT Registry. Dermatol. Ther..

[19] Garcia-Doval I., Descalzo M.A., Mason K.J., Cohen A.D., Ormerod A.D., Gómez-García F.J., Cazzaniga S., Feldhamer I., H Ali H., Herrera-Acosta E. (2018). Cumulative exposure to biological therapy and risk of cancer in patients with psoriasis: A meta-analysis of Psonet studies from Israel, Italy, Spain, the U.K. and Republic of Ireland. Br. J. Dermatol..

[20] Askling J., van Vollenhoven R.F., Granath F., Raaschou P., Fored C.M., Baecklund E., Dackhammar C., Feltelius N., Cöster L., Geborek P. (2009). Cancer risk in patients with rheumatoid arthritis treated with anti–tumor necrosis factor α therapies: Does the risk change with the time since start of treatment?. Arthritis Rheum..

[21] Gossec L., Siebert S., Bergmans P., de Vlam K., Gremese E., Joven-Ibáñez B., Korotaeva T.V., Lavie F., Noël W., Nurmohamed M.T. (2023). Long-term effectiveness and persistence of ustekinumab and TNF inhibitors in patients with psoriatic arthritis: Final 3-year results from the PsABio real-world study. Ann. Rheum. Dis..

[22] Long A., Rahmaoui A., Rothman K.J., Guinan E., Eisner M., Bradley M.S., Iribarren C., Chen H., Carrigan G., Rosén K. (2014). Incidence of malignancy in patients with moderate-to-severe asthma treated with or without omalizumab. J. Allergy Clin. Immunol..

[23] Wadström H., Frisell T., Askling J., Group ftA-RTiSS (2017). Malignant Neoplasms in Patients with Rheumatoid Arthritis Treated with Tumor Necrosis Factor Inhibitors, Tocilizumab, Abatacept, or Rituximab in Clinical Practice: A Nationwide Cohort Study From Sweden. JAMA Intern. Med..

[24] Andersen N.N., Pasternak B., Basit S., Andersson M., Svanström H., Caspersen S., Munkholm P., Hviid A., Jess T. (2014). Association between tumor necrosis factor-α antagonists and risk of cancer in patients with inflammatory bowel disease. JAMA.

[25] Lebwohl M.G., Koo J.Y., Armstrong A.W., Strober B.E., Yoon S.H., Rawnsley N.N., Goehring E.L., Jacobson A.A. (2025). Brodalumab: Seven-Year US Pharmacovigilance Report. Dermatol. Ther..

[26] Molina-Collada J., Otero-Varela L., Vela P., Manrique S., Erausquin C., Campos C., Manero J., Calvo J., Expósito L., González J.G. (2026). Cancer risk in patients with rheumatoid arthritis receiving biologic and targeted synthetic disease-modifying antirheumatic drugs: Results from the BIOBADASER III registry. Rheumatology.

[27] Song J., Kim S.R., Kim Y.J., Park S.J., Jeong S., Park S.M. (2025). Risk of hematologic malignancies in psoriasis and rheumatoid arthritis patients using long term TNF-α inhibitors: A retrospective nationwide study. Sci. Rep..

[28] Morel J., Wetzman A., Wendling D., Soubrier M., Hoang S., Briançon D., Roth O., Goupille P., Gottenberg J.E., Xavier M. (2025). Risk of cancer in patients with rheumatoid arthritis under tocilizumab: Data from the French national registry REGATE. Jt. Bone Spine.

[29] Huss V., Bower H., Hellgren K., Frisell T., Askling J. (2023). Cancer risks with JAKi and biological disease-modifying antirheumatic drugs in patients with rheumatoid arthritis or psoriatic arthritis: A national real-world cohort study. Ann. Rheum. Dis..

[30] Ma K.S., Brumbaugh B., Saff R.R., Phipatanakul W., Tsai S.Y., Westmeijer M., Holt A., Ebriani J., Camargo C.A., Chen S.T. (2025). Dupilumab and lymphoma risk among patients with asthma: A population-based cohort study. Eur. Respir. J..

[31] Westermann R., Cordtz R.L., Duch K., Mellemkjaer L., Hetland M.L., Magnussen B., Dreyer L. (2025). Cancer risk with tocilizumab/sarilumab, abatacept and rituximab treatment in patients with rheumatoid arthritis: A Danish cohort study. Rheumatology.

[32] Kridin K., Abdelghaffar M., Mruwat N., Ludwig R.J., Thaçi D. (2024). Are interleukin 17 and interleukin 23 inhibitors associated with malignancies?-Insights from an international population-based study. J. Eur. Acad. Dermatol. Venereol..

[33] Calzari P., Chiei Gallo A., Barei F., Bono E., Cugno M., Marzano A.V., Ferrucci S.M. (2024). Omalizumab for the Treatment of Chronic Spontaneous Urticaria in Adults and Adolescents: An Eight-Year Real-Life Experience. J. Clin. Med..

[34] Chaparro M., Baston-Rey I., Fernández-Salgado E., García J.G., Ramos L., Diz-Lois Palomares M.T., Argüelles-Arias F., Flores E.I., Cabello M., Iturria S.R. (2022). Long-Term Real-World Effectiveness and Safety of Ustekinumab in Crohn’s Disease Patients: The SUSTAIN Study. Inflamm. Bowel Dis..

[35] Blauvelt A., Thaçi D., Papp K.A., Ho V., Ghoreschi K., Kim B.S., Miller M., Shen Y.K., You Y., Chan D. (2023). Safety of guselkumab in patients with psoriasis with a history of malignancy: 5-year results from the VOYAGE 1 and VOYAGE 2 trials. Br. J. Dermatol..

[36] Gottlieb A.B., Deodhar A., McInnes I.B., Baraliakos X., Reich K., Schreiber S., Bao W., Marfo K., Richards H.B., Pricop L. (2022). Long-term Safety of Secukinumab over Five Years in Patients with Moderate-to-Severe Plaque Psoriasis, Psoriatic Arthritis and Ankylosing Spondylitis: Update on Integrated Pooled Clinical Trial and Post-marketing Surveillance Data. Acta Derm.-Venereol..

[37] Warren R.B., Lebwohl M., Thaçi D., Gooderham M., Pinter A., Paul C., Gisondi P., Szilagyi B., White K., Deherder D. (2025). Bimekizumab efficacy and safety through 3 years in patients with moderate-to-severe plaque psoriasis: Long-term results from the BE RADIANT phase IIIb trial open-label extension period. Br. J. Dermatol..

[38] Mease P.J., Gensler L.S., Orbai A.M., Warren R.B., Bajracharya R., Ink B., Marten A., Massow U., Shende V., Manente M. (2025). Long-term safety of bimekizumab in adult patients with axial spondyloarthritis or psoriatic arthritis: Pooled results from integrated phase IIb/III clinical studies. RMD Open.

[39] Merola J.F., Papp K.A., Deodhar A., Blauvelt A., Kronbergs A., McDonald M.F., Eberhart N., Zhu D., Inman E., Grace E. (2025). Ixekizumab and Malignant Neoplasms: A Pooled Analysis of Data from 25 Randomized Clinical Trials. JAMA Dermatol..

[40] Sands B.E., D’Haens G., Clemow D.B., Irving P.M., Johns J.T., Gibble T.H., Abreu M.T., Lee S.D., Hisamatsu T., Kobayashi T. (2025). Three-Year Efficacy and Safety of Mirikizumab Following 152 Weeks of Continuous Treatment for Ulcerative Colitis: Results From the LUCENT-3 Open-Label Extension Study. Inflamm. Bowel Dis..

[41] Ferrante M., Panaccione R., Colombel J.F., Dubinsky M., Hisamatsu T., Lindsay J.O., Song A., Neimark E., Zhang Y., Kligys K. (2024). DOP53 Long-term Efficacy and Safety of Risankizumab in Patients with Moderate to Severe Crohn’s Disease up to 3 Years of Treatment: Results From the FORTIFY Open-Label Long-term Extension. J. Crohn’s Colitis.

[42] Panaccione R., Feagan B.G., Afzali A., Rubin D.T., Reinisch W., Panés J., Danese S., Hisamatsu T., A Terry N., Salese L. (2025). Efficacy and safety of intravenous induction and subcutaneous maintenance therapy with guselkumab for patients with Crohn’s disease (GALAXI-2 and GALAXI-3): 48-week results from two phase 3, randomised, placebo and active comparator-controlled, double-blind, triple-dummy trials. Lancet.

[43] Osterman M.T., Sandborn W.J., Colombel J.F., Robinson A.M., Lau W., Huang B., Pollack P.F., Thakkar R.B., Lewis J.D. (2014). Increased risk of malignancy with adalimumab combination therapy, compared with monotherapy, for Crohn’s disease. Gastroenterology.

[44] Sandborn W.J., Rutgeerts P., Gasink C., Jacobstein D., Zou B., Johanns J., Sands B.E., Hanauer S.B., Targan S., Ghosh S. (2018). Long-term efficacy and safety of ustekinumab for Crohn’s disease through the second year of therapy. Aliment. Pharmacol. Ther..

[45] Ghosh S., Feagan B.G., Ott E., Gasink C., Godwin B., Marano C., Miao Y., Ma T., Loftus E.V., Sandborn W.J. (2024). Safety of Ustekinumab in Inflammatory Bowel Disease: Pooled Safety Analysis Through 5 Years in Crohn’s Disease and 4 Years in Ulcerative Colitis. J. Crohn’s Colitis.

[46] Schiff M.H., Kremer J.M., Jahreis A., Vernon E., Isaacs J.D., van Vollenhoven R.F. (2011). Integrated safety in tocilizumab clinical trials. Arthritis Res. Ther..

[47] Simpson E.L., Merola J.F., Silverberg J.I., Reich K., Warren R.B., Staumont-Sallé D., Girolomoni G., Papp K., Bruin-Weller M., Thyssen J.P. (2022). Safety of tralokinumab in adult patients with moderate-to-severe atopic dermatitis: Pooled analysis of five randomized, double-blind, placebo-controlled phase II and phase III trials*. Br. J. Dermatol..

[48] Thaçi D., LSimpson E., Deleuran M., Kataoka Y., Chen Z., Gadkari A., Eckert L., Akinlade B., Graham N.M.H., Pirozzi G. (2019). Efficacy and safety of dupilumab monotherapy in adults with moderate-to-severe atopic dermatitis: A pooled analysis of two phase 3 randomized trials (LIBERTY AD SOLO 1 and LIBERTY AD SOLO 2). J. Dermatol. Sci..

[49] Langley R.G., Gherardi G., Coleman A., Ardeleanu M., Rodríguez-Marco A., Levy S., Bansal A., Chen Z., Rossi A.B., Shumel B. (2025). The Safety Data of Dupilumab for the Treatment of Moderate-to-Severe Atopic Dermatitis in Infants, Children, Adolescents, and Adults. Am. J. Clin. Dermatol..

[50] Bourdin A., Chupp G., Jackson D.J., Cohen D., Emerath U., Shavit A., Kurdyukova Y., Menzies-Gow A. (2024). MELTEMI and COLUMBA: 5-Year Comparative Safety Analysis of Benralizumab and Mepolizumab. J. Allergy Clin. Immunol. Pract..

[51] Busse W., Buhl R., Vidaurre C.F., Blogg M., Zhu J., Eisner M.D., Canvin J. (2012). Omalizumab and the risk of malignancy: Results from a pooled analysis. J. Allergy Clin. Immunol..

[52] Sandborn W.J., Lawendy N., Danese S., Su C., Loftus E.V., Hart A., Dotan I., Damião A.O.M.C., Judd D.T., Guo X. (2022). Safety and efficacy of tofacitinib for treatment of ulcerative colitis: Final analysis of OCTAVE Open, an open-label, long-term extension study with up to 7.0 years of treatment. Aliment. Pharmacol. Ther..

[53] Patel R.V., Clark L.N., Lebwohl M., Weinberg J.M. (2009). Treatments for psoriasis and the risk of malignancy. J. Am. Acad. Dermatol..

[54] Peleva E., Exton L.S., Kelley K., Kleyn C.E., Mason K.J., Smith C.H. (2018). Risk of cancer in patients with psoriasis on biological therapies: A systematic review. Br. J. Dermatol..

[55] Pouplard C., Brenaut E., Horreau C., Barnetche T., Misery L., Richard M.A., Aractingi S., Aubin F., Cribier B., Joly P. (2013). Risk of cancer in psoriasis: A systematic review and meta-analysis of epidemiological studies. J. Eur. Acad. Dermatol. Venereol..

[56] Kimball A.B., Schenfeld J., Accortt N.A., Anthony M.S., Rothman K.J., Pariser D. (2015). Cohort study of malignancies and hospitalized infectious events in treated and untreated patients with psoriasis and a general population in the United States. Br. J. Dermatol..

[57] Torre L.A., Bray F., Siegel R.L., Ferlay J., Lortet-Tieulent J., Jemal A. (2015). Global cancer statistics, 2012. CA Cancer J. Clin..

[58] Naldi L. (2010). Malignancy concerns with psoriasis treatments using phototherapy, methotrexate, cyclosporin, and biologics: Facts and controversies. Clin. Dermatol..

[59] Richard M.A., Barnetche T., Horreau C., Brenaut E., Pouplard C., Aractingi S., Aubin F., Cribier B., Joly P., Jullien D. (2013). Psoriasis, cardiovascular events, cancer risk and alcohol use: Evidence-based recommendations based on systematic review and expert opinion. J. Eur. Acad. Dermatol. Venereol..

[60] Archier E., Devaux S., Castela E., Gallini A., Aubin F., Le Maître M., Aractingi S., Bachelez H., Cribier B., Joly P. (2012). Carcinogenic risks of psoralen UV-A therapy and narrowband UV-B therapy in chronic plaque psoriasis: A systematic literature review. J. Eur. Acad. Dermatol. Venereol..

[61] Cappelli L.C., Shah A.A. (2020). The relationships between cancer and autoimmune rheumatic diseases. Best Pract. Res. Clin. Rheumatol..

[62] Franks A.L., Slansky J.E. (2012). Multiple associations between a broad spectrum of autoimmune diseases, chronic inflammatory diseases and cancer. Anticancer. Res..

[63] Bernal-Bello D., Frutos-Pérez B., Duarte-Millán M., Toledano-Macías M., Jaenes-Barrios B., Morales-Ortega A. (2025). Cancer Risk in Autoimmune and Immune-Mediated Diseases: A Narrative Review for Practising Clinicians. J. Clin. Med..

[64] Simon T.A., Thompson A., Gandhi K.K., Hochberg M.C., Suissa S. (2015). Incidence of malignancy in adult patients with rheumatoid arthritis: A meta-analysis. Arthritis Res. Ther..

[65] Bernatsky S., Ramsey-Goldman R., Labrecque J., Joseph L., Boivin J.F., Petri M., Zoma A., Manzi S., Urowitz M.B., Gladman D. (2013). Cancer risk in systemic lupus: An updated international multi-centre cohort study. J. Autoimmun..

[66] Onishi A., Sugiyama D., Kumagai S., Morinobu A. (2013). Cancer incidence in systemic sclerosis: Meta-analysis of population-based cohort studies. Arthritis Rheum..

[67] Yang Z., Lin F., Qin B., Liang Y., Zhong R. (2015). Polymyositis/dermatomyositis and malignancy risk: A metaanalysis study. J. Rheumatol..

[68] Liang Y., Yang Z., Qin B., Zhong R. (2014). Primary Sjogren’s syndrome and malignancy risk: A systematic review and meta-analysis. Ann. Rheum. Dis..

[69] Olén O., Erichsen R., Sachs M.C., Pedersen L., Halfvarson J., Askling J., Ekbom A., Sørensen H.T., Ludvigsson J.F. (2020). Colorectal cancer in ulcerative colitis: A Scandinavian population-based cohort study. Lancet.

[70] Beaugerie L., Itzkowitz S.H. (2015). Cancers complicating inflammatory bowel disease. N. Engl. J. Med..

[71] Jess T., Simonsen J., Jørgensen K.T., Pedersen B.V., Nielsen N.M., Frisch M. (2012). Decreasing risk of colorectal cancer in patients with inflammatory bowel disease over 30 years. Gastroenterology.

[72] Jess T., Loftus E.V., Velayos F.S., Harmsen W.S., Zinsmeister A.R., Smyrk T.C., Schleck C.D., Tremaine W.J., Melton J.L., Munkholm P. (2006). Risk of intestinal cancer in inflammatory bowel disease: A population-based study from olmsted county, Minnesota. Gastroenterology.

[73] Ekbom A., Helmick C., Zack M., Adami H.O. (1990). Increased risk of large-bowel cancer in Crohn’s disease with colonic involvement. Lancet.

[74] Maykel J.A., Hagerman G., Mellgren A.F., Li S.Y., Alavi K., Baxter N.N., Madoff R.D. (2006). Crohn’s colitis: The incidence of dysplasia and adenocarcinoma in surgical patients. Dis. Colon Rectum.

[75] Friedman S., Rubin P.H., Bodian C., Harpaz N., Present D.H. (2008). Screening and surveillance colonoscopy in chronic Crohn’s colitis: Results of a surveillance program spanning 25 years. Clin. Gastroenterol. Hepatol..

[76] Hemminki K., Li X., Sundquist J., Sundquist K. (2009). Cancer risks in Crohn disease patients. Ann. Oncol..

[77] Wang L.H., Yang Y.J., Cheng W.C., Wang W.M., Lin S.H., Shieh C.C. (2016). Higher Risk for Hematological Malignancies in Inflammatory Bowel Disease: A Nationwide Population-based Study in Taiwan. Am. J. Gastroenterol..

[78] Woo A., Lee S.W., Koh H.Y., Kim M.A., Han M.Y., Yon D.K. (2021). Incidence of cancer after asthma development: 2 independent population-based cohort studies. J. Allergy Clin. Immunol..

[79] Salameh L., Mahboub B., Khamis A., Alsharhan M., Tirmazy S.H., Dairi Y., Hamid Q., Hamoudi R., Al Heialy S. (2021). Asthma severity as a contributing factor to cancer incidence: A cohort study. PLoS ONE.

[80] Huang Q., Huang Y., Xu S., Yuan X., Liu X., Chen Z. (2024). Association of asthma and lung cancer risk: A pool of cohort studies and Mendelian randomization analysis. Medicine.

[81] Guo Y., Bian J., Chen Z., Fishe J.N., Zhang D., Braithwaite D., George T.J., Shenkman E.A., Licht J.D. (2023). Cancer incidence after asthma diagnosis: Evidence from a large clinical research network in the United States. Cancer Med..

[82] Qu Y.L., Liu J., Zhang L.X., Wu C.M., Chu A.J., Wen B.L., Ma C., Yan X.Y., Zhang X., Wang D.M. (2017). Asthma and the risk of lung cancer: A meta-analysis. Oncotarget.

[83] Jiang L., Sun Y.Q., Langhammer A., Brumpton B.M., Chen Y., Nilsen T.I., Leivseth L., Wahl S.G.F., Mai X.M. (2021). Asthma and asthma symptom control in relation to incidence of lung cancer in the HUNT study. Sci. Rep..

[84] Ye L., Wang F., Wu H., Yuan Y., Zhang Q. (2024). Evidence of the association between asthma and lung cancer risk from mendelian randomization analysis. Sci. Rep..

[85] Merrill R.M., Isakson R.T., Beck R.E. (2007). The association between allergies and cancer: What is currently known?. Ann. Allergy Asthma Immunol..

[86] Azad N., Rojanasakul Y., Vallyathan V. (2008). Inflammation and lung cancer: Roles of reactive oxygen/nitrogen species. J. Toxicol. Environ. Health B Crit. Rev..

[87] Kantor E.D., Hsu M., Du M., Signorello L.B. (2019). Allergies and Asthma in Relation to Cancer Risk. Cancer Epidemiol. Biomark. Prev..

[88] Rahman H.H., Niemann D., Munson-McGee S.H. (2023). Association between asthma, chronic bronchitis, emphysema, chronic obstructive pulmonary disease, and lung cancer in the US population. Environ. Sci. Pollut. Res..

[89] Liu X., Hemminki K., Försti A., Sundquist J., Sundquist K., Ji J. (2015). Cancer risk and mortality in asthma patients: A Swedish national cohort study. Acta Oncol..

[90] Fang H., Dong T., Li S., Zhang Y., Han Z., Liu M., Dong W., Hong Z., Fu M., Zhang H. (2023). A Bibliometric Analysis of Comorbidity of COPD and Lung Cancer: Research Status and Future Directions. Int. J. Chronic Obstr. Pulm. Dis..

[91] Zitvogel L., Tesniere A., Kroemer G. (2006). Cancer despite immunosurveillance: Immunoselection and immunosubversion. Nat. Rev. Immunol..

[92] Münz C., Moormann A. (2008). Immune escape by Epstein-Barr virus associated malignancies. Semin. Cancer Biol..

[93] O’Donovan P., Perrett C.M., Zhang X., Montaner B., Xu Y.-Z., Harwood C.A., McGregor J.M., Walker S.L., Hanaoka F., Karran P. (2005). Azathioprine and UVA light generate mutagenic oxidative DNA damage. Science.

[94] Vaengebjerg S., Skov L., Egeberg A., Loft N.D. (2020). Prevalence, Incidence, and Risk of Cancer in Patients with Psoriasis and Psoriatic Arthritis: A Systematic Review and Meta-analysis. JAMA Dermatol..

[95] Fiorentino D., Ho V., Lebwohl M.G., Leite L., Hopkins L., Galindo C., Goyal K., Langholff W., Fakharzadeh S., Srivastava B. (2017). Risk of malignancy with systemic psoriasis treatment in the Psoriasis Longitudinal Assessment Registry. J. Am. Acad. Dermatol..

[96] Nast A., Smith C., Spuls P.I., Avila Valle G., Bata-Csörgö Z., Boonen H., Jong E.D., Garcia-Doval I., Gisondi P., Kaur-Knudsen D. (2021). EuroGuiDerm Guideline on the systemic treatment of Psoriasis vulgaris—Part 2: Specific clinical and comorbid situations. J. Eur. Acad. Dermatol. Venereol..

[97] Elmets C.A., Leonardi C.L., Davis D.M., Gelfand J.M., Lichten J., Mehta N.N., Armstrong A.W., Connor C., Cordoro K.M., Elewski B.E. (2019). Joint AAD-NPF guidelines of care for the management and treatment of psoriasis with biologics. J. Am. Acad. Dermatol..

[98] Harrison M.L., Obermueller E., Maisey N.R., Hoare S., Edmonds K., Li N.F., Chao D., Hall K., Chooi L., Timotheadou E. (2007). Tumor necrosis factor alpha as a new target for renal cell carcinoma: Two sequential phase II trials of infliximab at standard and high dose. J. Clin. Oncol..

[99] Ioannidis J.P., Zhou Y., Chang C.Q., Schully S.D., Khoury M.J., Freedman A.N. (2014). Potential increased risk of cancer from commonly used medications: An umbrella review of meta-analyses. Ann. Oncol..

[100] Beaugerie L., Brousse N., Bouvier A.M., Colombel J.F., Lémann M., Cosnes J., Hébuterne X., Cortot A., Bouhnik Y., Gendre J.P. (2009). Lymphoproliferative disorders in patients receiving thiopurines for inflammatory bowel disease: A prospective observational cohort study. Lancet.

[101] Gordon H., Biancone L., Fiorino G., Katsanos K.H., Kopylov U., Al Sulais E., Axelrad J.E., Balendran K., Burisch J., De Ridder L. (2023). ECCO Guidelines on Inflammatory Bowel Disease and Malignancies. J. Crohn’s Colitis.

[102] Peyrin–Biroulet L., Deltenre P., de Suray N., Branche J., Sandborn W.J., Colombel J.F. (2008). Efficacy and Safety of Tumor Necrosis Factor Antagonists in Crohn’s Disease: Meta-Analysis of Placebo-Controlled Trials. Clin. Gastroenterol. Hepatol..

[103] Axelrad J., Bernheim O., Colombel J.F., Malerba S., Ananthakrishnan A., Yajnik V., Hoffman G., Agrawal M., Lukin D., Desai A. (2016). Risk of New or Recurrent Cancer in Patients with Inflammatory Bowel Disease and Previous Cancer Exposed to Immunosuppressive and Anti-Tumor Necrosis Factor Agents. Clin. Gastroenterol. Hepatol..

[104] Williams C.J., Peyrin-Biroulet L., Ford A.C. (2014). Systematic review with meta-analysis: Malignancies with anti-tumour necrosis factor-α therapy in inflammatory bowel disease. Aliment. Pharmacol. Ther..

[105] Axelrad J.E., Hashash J.G., Itzkowitz S.H. (2024). AGA Clinical Practice Update on Management of Inflammatory Bowel Disease in Patients with Malignancy: Commentary. Clin. Gastroenterol. Hepatol..

[106] Kafel A.J., Muzalyova A., Schnoy E. (2025). Malignancy and Inflammatory Bowel Disease (IBD): Incidence and Prevalence of Malignancy in Correlation to IBD Therapy and Disease Activity-A Retrospective Cohort Analysis over 5 Years. Biomedicines.

[107] Carsons S. (1997). The association of malignancy with rheumatic and connective tissue diseases. Semin. Oncol..

[108] Sebbag E., Lauper K., Molina-Collada J., Aletaha D., Askling J., Gente K., Bertheussen H., Bitoun S., Bolek E.C., Burmester G.R. (2024). 2024 EULAR points to consider on the initiation of targeted therapies in patients with inflammatory arthritis and a history of cancer. Ann. Rheum. Dis..

[109] Kerschbaumer A., Smolen J.S., Dougados M., de Wit M., Primdahl J., McInnes I., van Der Heijde D., Baraliakos X., Falzon L., Gossec L. (2020). Pharmacological treatment of psoriatic arthritis: A systematic literature research for the 2019 update of the EULAR recommendations for the management of psoriatic arthritis. Ann. Rheum. Dis..

[110] Askling J., Fored C.M., Baecklund E., Brandt L., Backlin C., Ekbom A., Heijde D., Baraliakos X., Falzon L., Gossec L. (2005). Haematopoietic malignancies in rheumatoid arthritis: Lymphoma risk and characteristics after exposure to tumour necrosis factor antagonists. Ann. Rheum. Dis..

[111] Bongartz T., Sutton A.J., Sweeting M.J., Buchan I., Matteson E.L., Montori V. (2006). Anti-TNF antibody therapy in rheumatoid arthritis and the risk of serious infections and malignancies: Systematic review and meta-analysis of rare harmful effects in randomized controlled trials. JAMA.

[112] Mariette X., Matucci-Cerinic M., Pavelka K., Taylor P., van Vollenhoven R., Heatley R., Walsh C., Lawson R., Reynolds A., Emery P. (2011). Malignancies associated with tumour necrosis factor inhibitors in registries and prospective observational studies: A systematic review and meta-analysis. Ann. Rheum. Dis..

[113] Wolfe F., Michaud K. (2007). Biologic treatment of rheumatoid arthritis and the risk of malignancy: Analyses from a large US observational study. Arthritis Rheum..

[114] Dreyer L., Mellemkjær L., Andersen A.R., Bennett P., Poulsen U.E., Ellingsen T.J., Hansen T.H., Jensen D.V., Linde L., Lindegaard H.M. (2013). Incidences of overall and site specific cancers in TNFα inhibitor treated patients with rheumatoid arthritis and other arthritides—A follow-up study from the DANBIO Registry. Ann. Rheum. Dis..

[115] Driscoll C.B., Rich J.M., Isaacson D., Nicolas J., Jiang Y., Mi X., Yang C., Kocsuta V., Goh R., Patel N. (2025). Tumor Necrosis Factor-Alpha Inhibitor Use and Malignancy Risk: A Systematic Review and Patient Level Meta-Analysis. Cancers.

[116] Ytterberg S.R., Bhatt D.L., Mikuls T.R., Koch G.G., Fleischmann R., Rivas J.L., Germino R., Menon S., Sun Y., Wang C. (2022). Cardiovascular and Cancer Risk with Tofacitinib in Rheumatoid Arthritis. N. Engl. J. Med..

[117] Westermann R., Cordtz R., Duch K., Mellemkjaer L., Hetland M.L., Rasmussen L.A., Dreyer L. (2025). Cancer recurrence risk with bDMARD treatment in patients with rheumatoid arthritis and a history of cancer: A nationwide Danish register-based cohort study. RMD Open.

[118] Chyuan I.T., Lai J.H. (2020). New insights into the IL-12 and IL-23: From a molecular basis to clinical application in immune-mediated inflammation and cancers. Biochem. Pharmacol..

[119] Zhao J., Chen X., Herjan T., Li X. (2020). The role of interleukin-17 in tumor development and progression. J. Exp. Med..

[120] Jairath V., Acosta Felquer M.L., Cho R.J. (2024). IL-23 inhibition for chronic inflammatory disease. Lancet.

[121] Wu S., Xu Y., Yang L., Guo L., Jiang X. (2023). Short-term risk and long-term incidence rate of infection and malignancy with IL-17 and IL-23 inhibitors in adult patients with psoriasis and psoriatic arthritis: A systematic review and meta-analysis. Front. Immunol..

[122] Satolli F., Gerosa S., Burlando M., Cozzani E.C., Lasagni C., Manfredini M., Narcisi A., Marzano A.V., Carrera C.G., Megna M. (2025). Psoriasis vulgaris in patients with a recent history of neoplasia: Safety of interleukin-23 inhibitors. A multicentre retrospective study. Clin. Exp. Dermatol..

[123] Peyrin-Biroulet L., Chapman J.C., Colombel J.F., Caprioli F., D’Haens G., Ferrante M., Ferrante M., Schreiber S., Atreya R., Danese S. (2024). Risankizumab Versus Ustekinumab for Moderate-to-Severe Crohn’s Disease. N. Engl. J. Med..

[124] Sandborn W.J., Ferrante M., Bhandari B.R., Berliba E., Hibi T., D’Haens G.R., Tuttle J.L., Krueger K., Friedrich S., Durante M. (2022). Efficacy and Safety of Continued Treatment with Mirikizumab in a Phase 2 Trial of Patients with Ulcerative Colitis. Clin. Gastroenterol. Hepatol..

[125] Jaber F., Ayyad M., Alsakarneh S., Alsharaeh T., Salahat A.J., Jaber M., Gangwani M.K., Abboud Y., Mohamed I., Ali H. (2025). Efficacy and Safety of Interleukin-12/23 and Interleukin-23 Inhibitors for Ulcerative Colitis: A Systematic Review and Meta-Analysis of Randomized Controlled Trials. Am. J. Ther..

[126] Biskup L., Semeradt J., Rogowska J., Chort W., Durko Ł., Małecka-Wojciesko E. (2025). New Interleukin-23 Antagonists’ Use in Crohn’s Disease. Pharmaceuticals.

[127] Danese S., Panaccione R., Feagan B.G., Afzali A., Rubin D.T., Sands B.E., Reinisch W., Panés J., Sahoo A., Terry N.A. (2024). Efficacy and safety of 48 weeks of guselkumab for patients with Crohn’s disease: Maintenance results from the phase 2, randomised, double-blind GALAXI-1 trial. Lancet Gastroenterol. Hepatol..

[128] Wang S., Sun H., Wang Q., Xiao H. (2025). Efficacy and safety of IL-23 p19 inhibitors in the treatment for inflammatory bowel disease: A systematic review and meta-analysis. Front. Pharmacol..

[129] Singh S., Loftus E.V., Limketkai B.N., Haydek J.P., Agrawal M., Scott F.I., Ananthakrishnan A.N. (2024). AGA Clinical Guidelines Committee AGA Living Clinical Practice Guideline on Pharmacological Management of Moderate-to-Severe Ulcerative Colitis. Gastroenterology.

[130] Bilal J., Berlinberg A., Riaz I.B., Faridi W., Bhattacharjee S., Ortega G., Murad M.H., Wang Z., Prokop L.J., Alhifany A.A. (2019). Risk of Infections and Cancer in Patients with Rheumatologic Diseases Receiving Interleukin Inhibitors: A Systematic Review and Meta-analysis. JAMA Netw. Open.

[131] Ru Y., Ding X., Luo Y., Li H., Sun X., Zhou M., Zhou Y., Kuai L., Xing M., Liu L. (2021). Adverse Events Associated with Anti-IL-23 Agents: Clinical Evidence and Possible Mechanisms. Front. Immunol..

[132] Battista T., Gallo L., Martora F., Fattore D., Potestio L., Cacciapuoti S., Scalvenzi M., Megna M. (2024). Biological Therapy for Psoriasis in Cancer Patients: An 8-Year Retrospective Real-Life Study. J. Clin. Med..

[133] Schwarz C.W., Rasmussen M.K., Bertelsen T., Iversen L., Skov L., Loft N. (2025). Comparative Cancer Risk in Patients with Psoriasis Treated with Adalimumab, Secukinumab, or Ustekinumab. JAMA Dermatol..

[134] Papp K.A., Melosky B., Sehdev S., Hotte S.J., Beecker J.R., Kirchhof M.G., Turchin I., Dutz J.P., Gooderham M.J., Gniadecki R. (2023). Use of Systemic Therapies for Treatment of Psoriasis in Patients with a History of Treated Solid Tumours: Inference-Based Guidance from a Multidisciplinary Expert Panel. Dermatol. Ther..

[135] Gottlieb A.B., Kalb R.E., Langley R.G., Krueger G.G., de Jong E.M., Guenther L., Goyal K., Fakharzadeh S., Chevrier M., Calabro S. (2014). Safety observations in 12095 patients with psoriasis enrolled in an international registry (PSOLAR): Experience with infliximab and other systemic and biologic therapies. J. Drugs Dermatol. JDD.

[136] Lebwohl M., Deodhar A., Griffiths C.E.M., Menter M.A., Poddubnyy D., Bao W., Jehl V., Marfo K., Primatesta P., Shete A. (2021). The risk of malignancy in patients with secukinumab-treated psoriasis, psoriatic arthritis and ankylosing spondylitis: Analysis of clinical trial and postmarketing surveillance data with up to five years of follow-up. Br. J. Dermatol..

[137] Hueber W., Sands B.E., Lewitzky S., Vandemeulebroecke M., Reinisch W., Higgins P.D., Wehkamp J., Feagan B.G., Michael Yao D., Karczewski M. (2012). Secukinumab, a human anti-IL-17A monoclonal antibody, for moderate to severe Crohn’s disease: Unexpected results of a randomised, double-blind placebo-controlled trial. Gut.

[138] Ngiow S.F., Teng M.W., Smyth M.J. (2013). A balance of interleukin-12 and -23 in cancer. Trends Immunol..

[139] Gordon K.B., Papp K.A., Langley R.G., Ho V., Kimball A.B., Guzzo C., Yeilding N., Szapary P.O., Fakharzadeh S., Li S. (2012). Long-term safety experience of ustekinumab in patients with moderate to severe psoriasis (Part II of II): Results from analyses of infections and malignancy from pooled phase II and III clinical trials. J. Am. Acad. Dermatol..

[140] van Vollenhoven R.F., Hahn B.H., Tsokos G.C., Wagner C.L., Lipsky P., Touma Z., Werth V.P., Gordon R.M., Zhou B., Hsu B. (2018). Efficacy and safety of ustekinumab, an IL-12 and IL-23 inhibitor, in patients with active systemic lupus erythematosus: Results of a multicentre, double-blind, phase 2, randomised, controlled study. Lancet.

[141] Ghosh S., Gensler L.S., Yang Z., Gasink C., Chakravarty S.D., Farahi K., Ramachandran P., Elyssa Ott E., Strober B.E. (2019). Ustekinumab Safety in Psoriasis, Psoriatic Arthritis, and Crohn’s Disease: An Integrated Analysis of Phase II/III Clinical Development Programs. Drug Saf..

[142] Sandborn W.J., Rebuck R., Wang Y., Zou B., Adedokun O.J., Gasink C., Sands B.E., Hanauer S.B., Targan S., Ghosh S. (2022). Five-Year Efficacy and Safety of Ustekinumab Treatment in Crohn’s Disease: The IM-UNITI Trial. Clin. Gastroenterol. Hepatol..

[143] Solitano V., Ma C., Hanžel J., Panaccione R., Feagan B.G., Jairath V. (2023). Advanced Combination Treatment with Biologic Agents and Novel Small Molecule Drugs for Inflammatory Bowel Disease. Gastroenterol. Hepatol..

[144] Vuyyuru S.K., Solitano V., Hogan M., MacDonald J.K., Zayadi A., Parker C.E., Sands B.E., Panaccione R., Narula N., Feagan B.G. (2023). Efficacy and Safety of IL-12/23 and IL-23 Inhibitors for Crohn’s Disease: Systematic Review and Meta-Analysis. Dig. Dis. Sci..

[145] Hong S.J., Zenger C., Pecoriello J., Pang A., Vallely M., Hudesman D.P., Chang S., Axelrad J.E. (2022). Ustekinumab and Vedolizumab Are Not Associated with Subsequent Cancer in IBD Patients with Prior Malignancy. Inflamm. Bowel Dis..

[146] Ghasemi K., Ghasemi K. (2022). Evaluation of the Tocilizumab therapy in human cancers: Latest evidence and clinical potential. J. Clin. Pharm. Ther..

[147] Kim S.C., Pawar A., Desai R.J., Solomon D.H., Gale S., Bao M., Sarsour K., Schneeweiss S. (2019). Risk of malignancy associated with use of tocilizumab versus other biologics in patients with rheumatoid arthritis: A multi-database cohort study. Semin. Arthritis Rheum..

[148] Campbell L., Chen C., Bhagat S.S., Parker R.A., Östör A.J. (2011). Risk of adverse events including serious infections in rheumatoid arthritis patients treated with tocilizumab: A systematic literature review and meta-analysis of randomized controlled trials. Rheumatology.

[149] Luthra P., Peyrin-Biroulet L., Ford A.C. (2015). Systematic review and meta-analysis: Opportunistic infections and malignancies during treatment with anti-integrin antibodies in inflammatory bowel disease. Aliment. Pharmacol. Ther..

[150] Bonovas S., Fiorino G., Allocca M., Lytras T., Nikolopoulos G.K., Peyrin-Biroulet L., Danese S. (2016). Biologic Therapies and Risk of Infection and Malignancy in Patients with Inflammatory Bowel Disease: A Systematic Review and Network Meta-analysis. Clin. Gastroenterol. Hepatol..

[151] Ge W.S., Fan J.G. (2015). Integrin antagonists are effective and safe for Crohn’s disease: A meta-analysis. World J. Gastroenterol..

[152] Müller S., Maintz L., Bieber T. (2024). Treatment of atopic dermatitis: Recently approved drugs and advanced clinical development programs. Allergy.

[153] Beck L.A., Bissonnette R., Deleuran M., Nakahara T., Galus R., Coleman A., Gherardi G., Xiao J., Dingman R., Xu C. (2024). Dupilumab in Adults with Moderate to Severe Atopic Dermatitis: A 5-Year Open-Label Extension Study. JAMA Dermatol..

[154] Tanczosova M., Hugo J., Gkalpakiotis S. (2023). Treatment of Severe Atopic Dermatitis with Dupilumab in Patients with Advanced Cancer. J. Clin. Med..

[155] Ferreira C., Freitas E., Torres T. (2024). Efficacy and safety of dupilumab in a patient with metastatic clear cell renal cell carcinoma. J. Int. Med. Res..

[156] Zhang Y., Jing D., Cheng J., Chen X., Shen M., Liu H. (2022). The efficacy and safety of IL-13 inhibitors in atopic dermatitis: A systematic review and meta-analysis. Front. Immunol..

[157] Park A., Wong L., Lang A., Kraus C., Anderson N., Elsensohn A. (2023). Cutaneous T-cell lymphoma following dupilumab use: A systematic review. Int. J. Dermatol..

[158] Hasan I., Parsons L., Duran S., Zinn Z. (2024). Dupilumab therapy for atopic dermatitis is associated with increased risk of cutaneous T cell lymphoma: A retrospective cohort study. J. Am. Acad. Dermatol..

[159] Amatore F., Neildez M., de Masson A., Battistella M., Tauber M., Ingen-Housz-Oro S., Droitcourt C., Staumont-Sallé D., Beylot-Barry M. (2026). Cutaneous T-cell lymphomas and dupilumab for atopic dermatitis: A systematic review and expert consensus. J. Eur. Acad. Dermatol. Venereol..

[160] Formentini A., Braun P., Fricke H., Link K.H., Henne-Bruns D., Kornmann M. (2012). Expression of interleukin-4 and interleukin-13 and their receptors in colorectal cancer. Int. J. Color. Dis..

[161] Zaazouee M.S., Alwarraqi A.G., Mohammed Y.A., Badheeb M.A., Farhat A.M., Eleyan M., Morad A., Zeid M.A.A., Mohamed A.S., AbuEl-Enien H. (2022). Dupilumab efficacy and safety in patients with moderate to severe asthma: A systematic review and meta-analysis. Front. Pharmacol..

[162] Brusselle G.G., Koppelman G.H. (2022). Biologic Therapies for Severe Asthma. N. Engl. J. Med..

[163] Castro M., Corren J., Pavord I.D., Maspero J., Wenzel S., Rabe K.F., William W., Busse M.D., Ford L., Sher L. (2018). Dupilumab Efficacy and Safety in Moderate-to-Severe Uncontrolled Asthma. N. Engl. J. Med..

[164] Bhatt S.P., Rabe K.F., Hanania N.A., Vogelmeier C.F., Cole J., Bafadhel M., Christenson S.A., Papi A., Singh D., Laws E. (2023). Dupilumab for COPD with Type 2 Inflammation Indicated by Eosinophil Counts. N. Engl. J. Med..

[165] Gallagher A., Edwards M., Nair P., Drew S., Vyas A., Sharma R., Marsden P.A., Wang R., Evans D.J. (2021). Anti-interleukin-13 and anti-interleukin-4 agents versus placebo, anti-interleukin-5 or anti-immunoglobulin-E agents, for people with asthma. Cochrane Database Syst. Rev..

[166] Panettieri R.A., Sjöbring U., Péterffy A., Wessman P., Bowen K., Piper E., Colice G., Brightling C.E. (2018). Tralokinumab for severe, uncontrolled asthma (STRATOS 1 and STRATOS 2): Two randomised, double-blind, placebo-controlled, phase 3 clinical trials. Lancet Respir. Med..

[167] Corren J., Lemanske R.F., Hanania N.A., Korenblat P.E., Parsey M.V., Arron J.R., Harris J.M., Scheerens H., Wu L.C., Su Z. (2011). Lebrikizumab Treatment in Adults with Asthma. N. Engl. J. Med..

[168] Braddock M., Hanania N.A., Sharafkhaneh A., Colice G., Carlsson M. (2018). Potential Risks Related to Modulating Interleukin-13 and Interleukin-4 Signalling: A Systematic Review. Drug Saf..

[169] Farne H.A., Wilson A., Milan S., Banchoff E., Yang F., Powell C.V. (2022). Anti-IL-5 therapies for asthma. Cochrane Database Syst. Rev..

[170] Holguin F., Cardet J.C., Chung K.F., Diver S., Ferreira D.S., Fitzpatrick A., Gaga M., Kellermeyer L., Khurana S., Knight S. (2020). Management of severe asthma: A European Respiratory Society/American Thoracic Society guideline. Eur. Respir. J..

[171] Soegiharto R., Van der Wind E., Aghdam M.A., Sørensen J.A., Van Lindonk E., Demir F.B., Porras N.M., Matsuo Y., Kiefer L., Knulst A.C. (2025). Spectrum and Impact of Reported Side Effects of Omalizumab in Patients with Chronic Urticaria: A Long-Term Multicentre Real-World Study. Clin. Exp. Allergy.

[172] Tharp M.D., Bernstein J.A., Kavati A., Ortiz B., MacDonald K., Denhaerynck K., Abraham I., Lee C.S. (2019). Benefits and Harms of Omalizumab Treatment in Adolescent and Adult Patients with Chronic Idiopathic (Spontaneous) Urticaria: A Meta-analysis of “Real-world” Evidence. JAMA Dermatol..

[173] Kolkhir P., Bonnekoh H., Metz M., Maurer M. (2024). Chronic Spontaneous Urticaria: A Review. JAMA.

[174] Pongdee T., Li J.T. (2025). Omalizumab safety concerns. J. Allergy Clin. Immunol..

[175] Smith C.H., Jabbar-Lopez Z.K., Yiu Z.Z., Bale T., Burden A.D., Coates L.C., Cruickshank M., Hadoke T., MacMahon E., Murphy R. (2017). British Association of Dermatologists guidelines for biologic therapy for psoriasis 2017. Br. J. Dermatol..

[176] Lichtenstein G.R., Loftus E.V., Afzali A., Long M.D., Barnes E.L., Isaacs K.L., Ha C.Y. (2025). ACG Clinical Guideline: Management of Crohn’s Disease in Adults. Off. J. Am. Coll. Gastroenterol..

[177] Fraenkel L., Bathon J.M., England B.R., St Clair E.W., Arayssi T., Carandang K., Deane K.D., Genovese M., Huston K.K., Kerr G. (2021). 2021 American College of Rheumatology Guideline for the Treatment of Rheumatoid Arthritis. Arthritis Care Res..

[178] Bechman K., Song K., Abhishek A., Adas M., Ahmed A., Bray L., Davidson A., Deepak S., Dey M., Vere H.D. (2025). The 2025 British Society for Rheumatology guideline for the prescription and monitoring of conventional synthetic disease-modifying anti-rheumatic drugs. Rheumatology.

[179] Reddel H.K., Bacharier L.B., Bateman E.D., Brightling C.E., Brusselle G.G., Buhl R., Cruz A.A., Duijts L., Drazen J.M., FitzGerald J.M. (2022). Global Initiative for Asthma Strategy 2021: Executive Summary and Rationale for Key Changes. Am. J. Respir. Crit. Care Med..

[180] Halpin D.M.G., Masekela R., Vogelmeier C.F., Ozoh O.B., Cruz A.A., Reddel H.K., Yorgancioglu A., Agusti A., Boards of Directors of GOLD and GINA (2026). Addressing the global challenges of COPD and asthma: A shared vision from the Global Initiative for Chronic Obstructive Pulmonary Disease (GOLD) and the Global Initiative for Asthma (GINA). Eur. Respir. J..

[181] Chung K.F., Wenzel S.E., Brozek J.L., Bush A., Castro M., Sterk P.J., Adcock I.M., Bateman E.D., Bel E.H., Bleecker E.R. (2013). International ERS/ATS guidelines on definition, evaluation and treatment of severe asthma. Eur. Respir. J..

[182] British Thoracic Society (BTS), National Institute for Health and Care Excellence (NICE) (2025). BTS/NICE/SIGN joint guideline on asthma: Diagnosis, monitoring and chronic asthma management (November 2024)—Summary of recommendations. Thorax.

[183] Feuerstein J.D., Ho E.Y., Shmidt E., Singh H., Falck-Ytter Y., Sultan S., Terdiman J.P., AGA Institute Clinical Guidelines Committee (2021). AGA Clinical Practice Guidelines on the Medical Management of Moderate to Severe Luminal and Perianal Fistulizing Crohn’s Disease. Gastroenterology.

[184] Beaugerie L., Rahier J.F., Kirchgesner J. (2020). Predicting, Preventing, and Managing Treatment-Related Complications in Patients with Inflammatory Bowel Diseases. Clin. Gastroenterol. Hepatol..

[185] Blauvelt A., Papp K.A., Griffiths C.E., Randazzo B., Wasfi Y., Shen Y.K., Li S., Kimball A.B. (2017). Efficacy and safety of guselkumab, an anti-interleukin-23 monoclonal antibody, compared with adalimumab for the continuous treatment of patients with moderate to severe psoriasis: Results from the phase III, double-blinded, placebo- and active comparator-controlled VOYAGE 1 trial. J. Am. Acad. Dermatol..

[186] Feagan B.G., Rutgeerts P., Sands B.E., Hanauer S., Colombel J.F., Sandborn W.J., Assche G.V., Axler J., Kim H.J., Danese S. (2013). Vedolizumab as induction and maintenance therapy for ulcerative colitis. N. Engl. J. Med..

[187] Kundu R., Mukhopadhyay A., Kal N., Pal T.S., Sarkar N., Ball S., Maiti A., Mukherjee S. (2026). Global Epidemiologic Trends of Early-Onset Cancers From 1990 to 2021 and Projection to 2040. JCO Glob. Oncol..

[188] Bray F., Laversanne M., Sung H., Ferlay J., Siegel R.L., Soerjomataram I., Jemal A. (2024). Global cancer statistics 2022: GLOBOCAN estimates of incidence and mortality worldwide for 36 cancers in 185 countries. CA Cancer J. Clin..

[189] Finnegan P., Ahmad K., Sadlier M., Lynch M. (2023). A retrospective review of the management of patients following a malignancy diagnosis on biologic therapies for the treatment of dermatological disorders. JAAD Case Rep..

[190] Caballero C., Adam V., Birta O., Meheus L., Needham J., Hodgdon C., Gilhams L., Biurrun C., MacKenzie M., Spanic T. (2026). The Breast International Group (BIG) Patient Partnership: Embedding meaningful patient involvement in the design and conduct of breast cancer clinical research. Breast.

[191] Michaud S., Needham J., Sundquist S., Johnson D., Hanna S., Hosseinzadeh S., Bartekian V., Steele P., Benchimol S., Ross N. (2021). Patient and Patient Group Engagement in Cancer Clinical Trials: A Stakeholder Charter. Curr. Oncol..

[192] Görtz M., Brandl C., Nitschke A., Riediger A., Stromer D., Byczkowski M., Heuveline V., Weidemüller M. (2026). Digital twins for personalized treatment in uro-oncology in the era of artificial intelligence. Nat. Rev. Urol..

[193] Olawade D.B., Oisakede E.O., Bello O.J., Analikwu C.C., Egbon E., Ojo A. (2026). Digital twins in oncology: From predictive modelling to personalised treatment strategies. Crit. Rev. Oncol. Hematol..

[194] Görtz M. (2026). Digital twins: Past, present and future. Sci. Rep..

[195] Ratwani R.M., Sutton K., Galarraga J.E. (2024). Addressing AI Algorithmic Bias in Health Care. JAMA.

[196] O’Reilly S., Kathryn Carroll H., Murray D., Burke L., McCarthy T., O’Connor R., Kilty C., Lynch S., Feighan J., Cloherty M. (2023). Impact of the COVID-19 pandemic on cancer care in Ireland—Perspectives from a COVID-19 and Cancer Working Group. J. Cancer Policy.

[197] O’Reilly S., Luis I.V., Adam V., Razis E.D., Urruticoechea A., Arahmani A., Carrasco E., Chua B.H., Bliss J., Straehle C. (2025). Advancing equitable access to innovation in breast cancer. npj Breast Cancer.

